# RTF2 and CLASPIN localize CHK1 to the replication fork to control S-phase progression

**DOI:** 10.1126/sciadv.aec3174

**Published:** 2026-08-05

**Authors:** Cayla Broton, Catherine L. W. Miller, Penelope D. Ruiz, Nicolas J. Blobel, Yibing Yao, Brooke A. Conti, Ernst W. Schmid, Johannes C. Walter, Agata Smogorzewska

**Affiliations:** ^1^ Laboratory of Genome Maintenance, The Rockefeller University, New York, NY 10065, USA.; ^2^ Department of Biological Chemistry and Molecular Pharmacology, Blavatnik Institute, Harvard Medical School, Boston, MA 02115, USA.; ^3^ Howard Hughes Medical Institute, Boston, MA 02115, USA.

## Abstract

The essential kinase ataxia telangiectasia and Rad3-related (ATR) monitors the replicative (S) phase of the cell cycle to ensure faithful propagation of genetic material. While much is known about the mechanisms governing ATR activity at replication forks, how its effector checkpoint kinase 1 (CHK1) is regulated to create a localized hub of CHK1 activity at sites of replication remains poorly understood. Here, we report that replication termination factor 2 (RTF2), an essential replisome-associated protein, is necessary for maintaining CHK1 signaling at replication forks. RTF2 interacts with CHK1 and functions alongside the core replicative helicase complex-interacting protein CLASPIN to tether CHK1 to sites of DNA replication, controlling replication rates and preventing premature mitotic entry. These findings uncover how RTF2 and CLASPIN poise CHK1 at replication forks to facilitate its activation by ATR, creating fork-localized CHK1 activity that fuels unperturbed S-phase progression and promotes genome stability by maintaining the intrinsic S-G_2_ checkpoint.

## Teaser

Cells ensure that DNA is fully copied by anchoring CHK1, a control switch, to replication machinery via RTF2 and CLASPIN.

## INTRODUCTION

**Fig. 1.**
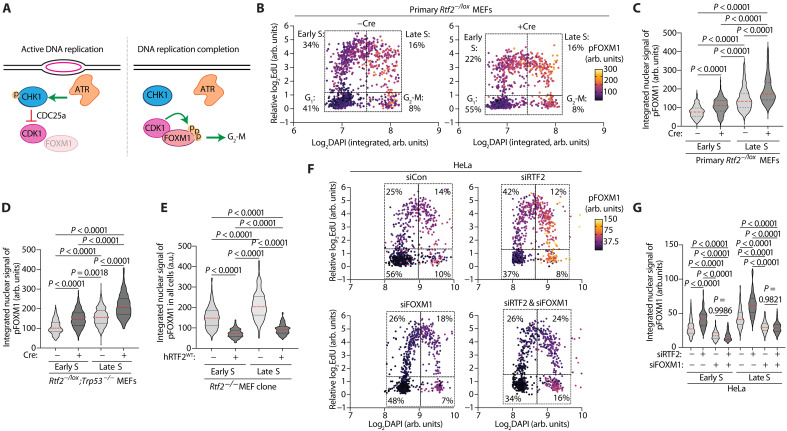
RTF2-deficient cells accumulate pFOXM1 while replication proceeds. (**A**) The intrinsic S-G_2_ checkpoint prevents mitotic entry while replication proceeds. ATR is activated at replication forks leading to downstream inhibition of CDK1 via CHK1 and suppression of the mitotic transcription program. (**B**) Representative quantitative image-based cytometry (QIBC) plots of primary *Rtf2^−/lox^* MEFs 72 hours after Cre. Nuclear pFOXM1 levels, the activated form of a mitotic transcription factor, are indicated using the color scale shown on the right. EdU incorporation indicates S-phase cells, and 4′,6-diamidino-2-phenylindole (DAPI) separates G_1_ and G_2_ populations. (**C**) Quantification of nuclear pFOXM1 levels across early versus late S phase of cells represented in (B). (**D**) Quantification of nuclear pFOXM1 levels across early versus late S phase of *Rtf2^−/lox^;Trp53*^−/−^ MEFs 72 hours after Cre as assessed by QIBC (corresponding plots shown in fig. S1B). (**E**) Quantification of nuclear pFOXM1 levels across early versus late S phase of *Rtf2^−/−^* MEF clone ± HA/Flag-hRTF2 as assessed by QIBC (corresponding plots shown in fig. S1C). (**F**) Representative pFOXM1 QIBC plots of HeLa cells 72 hours after transfection with indicated small interfering RNAs (siRNAs). (**G**) Quantification of nuclear pFOXM1 levels across early versus late S phase of cells represented in (F). Experiments conducted at least three times in biological replicates with consistent results for (B) to (G). Outliers removed with ROUT (1%) for (C) to (E) and (G). Significance evaluated by Kruskal-Wallis analysis of variance (ANOVA) with a Dunn’s posttest. a.u./arb. units, arbitrary units.

To ensure genomic stability, DNA replication must be largely completed before cells divide into two daughters. Cells sense ongoing unperturbed replication through basal activation of the intrinsic S-G_2_ checkpoint, which depends on the function of the essential ataxia telangiectasia and Rad3-related (ATR) kinase (*1*). Briefly, DNA replication leads to the transient exposure of small amounts of single-stranded DNA (ssDNA) that is coated by replication protein A (RPA), an abundant ssDNA binding protein. RPA-coated ssDNA is recognized by ATR-interacting protein and Ewing tumor-associated antigen 1 (ETAA1), leading to ATR recruitment and activation at the traveling replication fork (*2*–*7*). Ultimately, ATR phosphorylates and activates its essential effector kinase, checkpoint kinase 1 (CHK1). Once active, CHK1 phosphorylates its substrates, including cell division cycle 25a (CDC25a), a phosphatase necessary for maintaining the activation of cell cycle–dependent kinase 1 (CDK1). Phosphorylation by CHK1 targets CDC25a for degradation, suppressing CDK1 activity and the mitotic transcriptional program while replication proceeds (Fig. 1A) (*8*, *9*). Once DNA replication is complete, ssDNA is no longer exposed, and, therefore, ATR is not activated, leading to CDC25a accumulation and increased CDK1 activity that drives progression toward cell division.

Low-level activation of the ATR-CHK1 axis is also necessary during unperturbed replication to limit replication origin firing and to maintain replication fork speed (*10*–*14*). Loss or inhibition of ATR or CHK1 results in slow replication rates and increased levels of replication origin firing as revealed by DNA fiber and DNA combing experiments (*10*–*13*, *15*–*17*). The slow replication rate of CHK1-deficient cells appears to be downstream of excess origin firing as suppression of origin firing or supplementation with replication substrates can restore replication rates in CHK1-inhibited cells (*11*, *13*, *18*). Despite the clear importance of maintaining ATR-CHK1 activation during active replication, the mechanisms controlling the localized ATR-CHK1 activity at unperturbed replication forks remain incompletely understood.

Replication termination factor 2 (RTF2) is a highly conserved protein first identified as a component of the replisome that is regulated in response to replication stress by the proteasomal shuttle proteins DNA damage-inducible 1 and 2 (DDI1/2) (*19*). More recently, we have shown that RTF2 is an essential protein that regulates localization of the ribonuclease ribonuclease (RNase) H2 to replication forks to facilitate ribonucleotide excision and replication restart after stalling (*20*). RTF2-deficient cells were found to replicate DNA slowly, fire an excess of replication origins, and accumulate aberrant nuclear structures downstream of abnormal progression through mitosis (*20*). Despite these insights, the essential function of RTF2 at the unperturbed, progressing replication fork remains unknown.

Here, we show that RTF2-deficient cells bypass the intrinsic S-G_2_ checkpoint and exhibit slow replication fork progression downstream of excess replication origin firing. We identify an interaction between RTF2 and CHK1 and find that RTF2 and another human replisome component CLASPIN function to localize CHK1 to unperturbed replication forks, ensuring the low-level activation of CHK1 that limits origin firing, controls replication rates, and maintains the intrinsic S-G_2_ checkpoint.

## RESULTS

### RTF2-deficient cells bypass the intrinsic S-G_2_ checkpoint

Cells lacking RTF2 accumulate abnormal mitoses, micronuclei, and anaphase bridges that can be rescued with CDK1 inhibition (*20*). We hypothesized that this abnormal nuclear phenotype was the outcome of mitotic entry before the completion of replication. Consistent with this model, CDK1 inhibition resulted in RTF2-deficient cells having an elongated S phase, leading to completion of replication before cell division and normal nuclear morphology of daughter cells (*20*).

Entry into mitosis while replication proceeds is suppressed by the recently described intrinsic S-G_2_ checkpoint (Fig. 1A) (*8*). To assess whether RTF2-deficient cells bypass the S-G_2_ checkpoint, we first assessed accumulation of the phosphorylated, active form of the mitotic transcription factor forkhead box M1 (pFOXM1) in Cre-treated *Rtf2^−/lox^;Trp53^−/−^* mouse embryonic fibroblasts (MEFs) across S phase in thymidine-synchronized cells (fig. S1A). Cells lacking RTF2 began accumulating pFOXM1 signal in as little as 1 hour after release while RTF2-competent cells suppressed pFOXM1 accumulation for 4 to 5 hours postrelease.

Early–S-phase synchronization with thymidine has been found to drive changes in key cell cycle proteins like chromatin licensing and DNA replication factor 1 (CDT1), cyclin A, and geminin, potentially changing the dynamics of cell cycle progression (*21*). We thus turned to quantitative image-based cytometry (QIBC) to assess accumulation of pFOXM1 within individual cells of an asynchronous population (*22*). In Cre-treated primary *Rtf2^−/lox^* MEFs, we observed suppressed 5-Ethynyl-2′-deoxyuridine (EdU) incorporation characteristic of RTF2 loss along with increased levels of pFOXM1 signal across S phase as compared to control MEFs (Fig. 1, B and C). These results were confirmed in other RTF2-deficient cell lines, including *Rtf2^−/lox^;Trp53^−/−^* MEFs and an SV40-immortalized *Rtf2^−/−^* clone (Fig. 1, D and E; and fig. S1, B and C). HeLa cells depleted of RTF2 following transfection with small interfering RNA (siRNA) targeting RTF2 (siRTF2) similarly exhibited increased levels of nuclear pFOXM1 in replicating cells (Fig. 1, F and G). The pFOXM1 signal was abolished following transfection with siRNA targeting FOXM1 (siFOXM1), indicating specificity of the phospho-antibody (Fig. 1, F and G). Alongside accumulation of pFOXM1, RTF2-deficient cells were found to accumulate CYCLIN B1, the mitotic binding partner of CDK1, across S phase, further suggesting that the mitotic program is ineffectively suppressed in these cells (fig. S1, D to F).

**Fig. 2.**
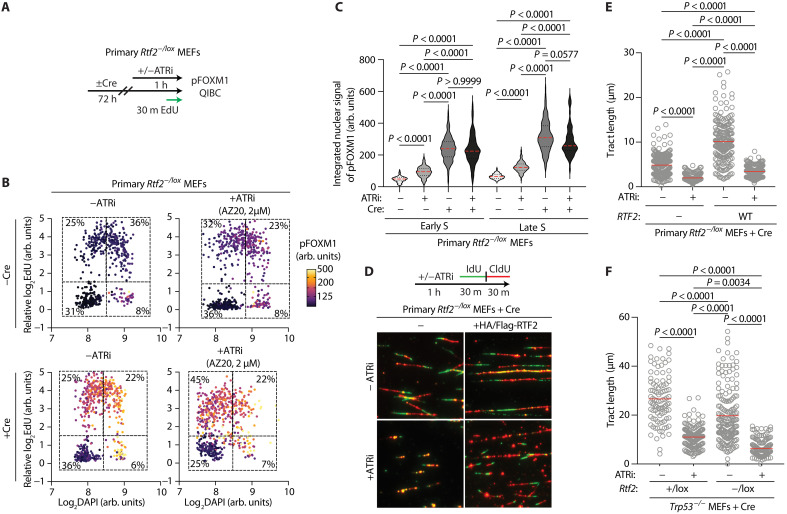
RTF2-deficient cells bypass the ATR-dependent intrinsic S-G_2_ checkpoint. (**A**) Treatment schematic for experiment shown in (B). h, hours; m, minutes. (**B**) Representative pFOXM1 QIBC plots of primary *Rtf2^−/lox^* MEFs 72 hours after Cre treated with ATR inhibitor (ATRi) AZ20 for 1 hour before fixation. (**C**) Quantification of nuclear pFOXM1 staining across early versus late S phase of cells represented in (B). arb. units, arbitrary units. (**D**) Top: ATRi treatment and labeling scheme for DNA fiber assay shown in (E) and DNA combing assay shown in (A). Bottom: Representative images of DNA fibers from experiment shown in (E). h, hours; m, minutes. (**E**) Quantification of replication tract lengths of primary *Rtf2^−/lox^* MEFs treated with ATRi 72 hours after Cre. (**F**) Quantification of replication tract lengths of *Rtf2^−/lox^;Trp53^−/−^* MEFs treated with ATRi 72 hours after Cre. Experiments conducted at least three times in biological replicates with consistent results for (B) to (F). Outliers removed with ROUT (1%) for (E) and (F). Significance evaluated by Kruskal-Wallis ANOVA with a Dunn’s posttest.

Depletion of ETAA1, which stimulates ATR activation following ssDNA accumulation within the S-G_2_ checkpoint, resulted in pFOXM1 accumulation in S-phase cells to similar levels as RTF2 depletion (fig. S2, A to C) (*4*–*8*). As reported, inhibition of ATR drove increased pFOXM1 accumulation during S phase in RTF2-competent MEFs (fig. S2D). Inhibition of other PIKK family kinases, ataxia-telangiectasia mutated (ATM) or DNA-dependent protein kinase, catalytic subunit (DNA-PKcs), did not induce pFOXM1 accumulation, indicating that the S-G_2_ checkpoint is dependent on ETAA1-stimulated ATR activity (fig. S2D). The ATR dependence of the checkpoint was confirmed in both primary and *Trp53^−/−^* RTF2-competent MEFs, which exhibited increased pFOXM1 accumulation upon acute ATR inhibition (Fig. 2, A to C; and fig. S2, D to G) (*8*). In RTF2-deficient cells, however, ATR inhibition did not further augment pFOXM1 levels in replicating cells. Similarly, acute inhibition of CHK1, ATR’s effector kinase, did not further increase pFOXM1 levels of late–S-phase RTF2-deficient cells (fig. S2, H and I). RTF2 loss drove higher levels of pFOXM1 than ATR inhibition, suggesting that acute treatment with ATR inhibitor may not fully inactivate the intrinsic S-G_2_ checkpoint, and it can be further suppressed directly or indirectly by loss of RTF2 under these conditions (Fig. 2, A to C; and fig. S2, E to G). Collectively, these data suggest that RTF2-deficient cells bypass the intrinsic S-G_2_ checkpoint, leading to premature CDK1 activity and pFOXM1accumulation while replication proceeds.

The cell cycle profiles of ATR-inhibited RTF2-deficient cells revealed an increased population of cells in mid–S phase with low levels of EdU incorporation, a finding indicative of aberrant S-phase progression (Fig. 2B and fig. S2F). We further assessed this phenotype by directly measuring DNA replication with DNA fiber and combing experiments, which allow for visualization of individual replication fork tracts, in Cre-treated primary *Rtf2^−/lox^* and *Rtf2^−/lox^*;*Trp53^−/−^* MEFs. ATR inhibition led to the formation of dual-labeled shortened tracts in RTF2-competent cells, likely due to increased origin firing, as previously reported (Fig. 2, D to F) (*10*). This phenotype was exacerbated in RTF2-deficient cells, resulting in very short replication tracts indicating extremely slow replication. These data are consistent with RTF2 having an ATR-independent function in the regulation of replication fork progression.

Previously, we have shown that RTF2 regulates the ribonuclease RNase H2 at replication forks to facilitate ribonucleotide excision and replication restart after stalling (*20*). We asked whether RNase H2 may similarly participate in S-G_2_ checkpoint maintenance, by assessing pFOXM1 accumulation in two independent RNase H2–deficient cell models. Loss of RNase H2, either by stable knockout of the catalytic RNase H2A (RH2A) subunit or by acute depletion of an ectopically expressed hemagglutinin-degradation tag-RH2A (HA-dTAG-RH2A), did not lead to increased levels of pFOXM1 across S phase (fig. S3) (*23*). These data confirm that RTF2’s function within the S-G_2_ checkpoint is independent of its impact on RNase H2 localization to the replication fork. Taken together, we conclude that RTF2 participates in the ATR-dependent intrinsic S-G_2_ checkpoint; however, the pleiotropic activities and the essential nature of these proteins, along with experimental limitations, preclude definitive epistasis analysis between them.

### RTF2- and TRESLIN-depleted cells exhibit distinct phenotypes of S-G_2_ checkpoint bypass

A recent report characterized premature entry into G_2_ following depletion of TRESLIN, an essential replication initiation factor that facilitates CDC45 recruitment (*24*). The authors suggested that TRESLIN upholds the S-G_2_ checkpoint to monitor DNA replication and prevent premature mitotic entry in an ATR-independent process. In light of this, we directly compared the mitotic S-G_2_ checkpoint bypass uncovered in RTF2-depleted cells to TRESLIN-depleted cells. While depletion of TRESLIN led to a notable increase in levels of pFOXM1 in S-phase cells, we observed distinct pFOXM1 profiles in TRESLIN-depleted and RTF2-depleted cells. Specifically, TRESLIN-depleted cells began to accumulate pFOXM1 in S phase much earlier than RTF2-deficient cells. TRESLIN-depleted cells accumulated pFOXM1 beginning in G_1_/early–S-phase cells, whereas RTF2-depleted cells only show increased levels of pFOXM1 in EdU-positive cells and across S phase (fig. S4).

While comparisons of phenotypes of essential proteins using partial depletions do not allow for mechanistic insight, our data suggest that TRESLIN and RTF2 maintain two separate layers of control of CDK1/2 activity. We hypothesize that, although total TRESLIN loss would preclude origin firing, partial TRESLIN depletion leads to a marked increase in origin firing at the very start of S phase driven by the previously reported increase in G_1_/early-S CDK1/2 activity in TRESLIN-depleted cells (*24*). While the cause of this enhanced CDK1/2 activity remains unclear, we propose that the large burden of activated forks depletes essential replication factors, potentially RTF2 or ATR, that are responsible for dampening CDK1 activity at the replication fork, leading to the observed early increase in pFOXM1 levels. We propose that TRESLIN is essential for controlling the number of properly licensed and fired replication origins during early S phase, whereas RTF2 functions at progressing replication forks to limit CDK1 activation. Although it is possible that phenotypes of RTF2 and TRESLIN loss in S phase may overlap, we have focused on understanding the changes occurring at RTF2-deficient replication forks that drive bypass of the ATR-dependent intrinsic S-G_2_ checkpoint.

### Unrestrained origin firing drives slow replication in RTF2-deficient cells

RTF2-deficient cells replicate DNA slowly and fire excess replication origins during an unperturbed S phase (*20*). We hypothesized that slow replication in RTF2-deficient cells was due to dysregulated origin firing, as seen in the setting of CHK1 depletion or loss (*11*–*14*).

**Fig. 3.**
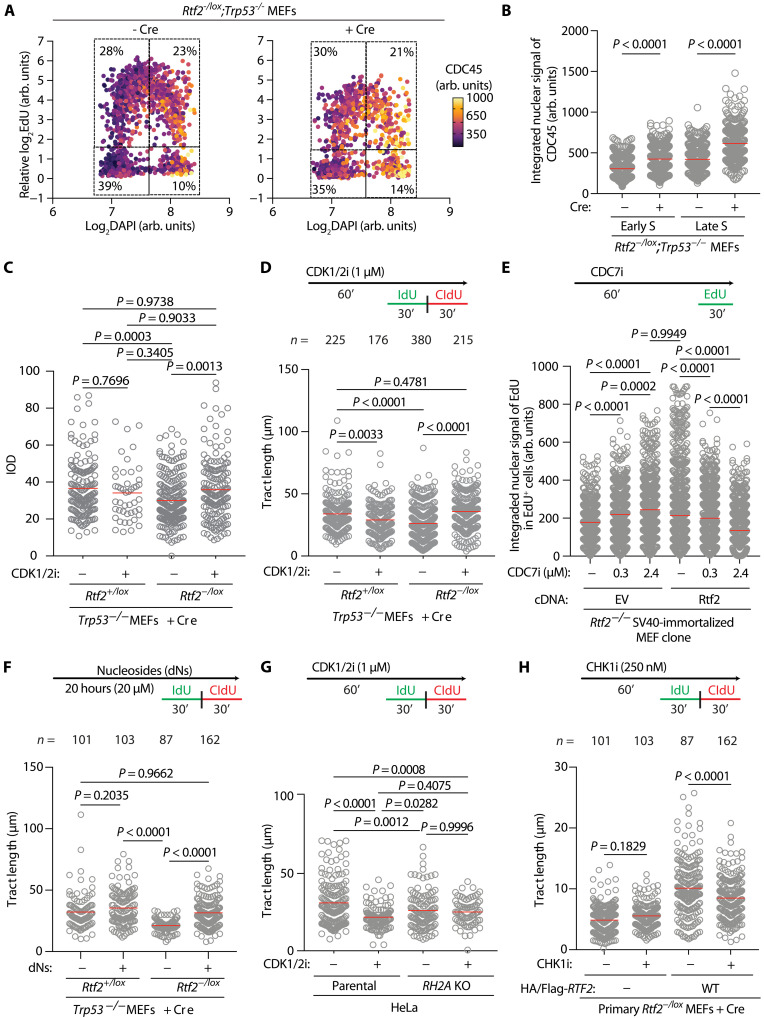
RTF2-CHK1 axis promotes unperturbed replication by limiting origin firing. (**A**) Representative chromatin-bound CDC45 QIBC of *Rtf2^−/lox^;Trp53^−/−^* MEFs 72 hours after Cre. (**B**) Integrated nuclear signal of chromatin-bound CDC45 in early– versus late–S-phase cells from (A). arb. units, arbitrary units. (**C**) Quantification of interorigin distances (IODs) from *Rtf2^+/lox^* versus *Rtf2^−/lox^Trp53^−/−^* MEFs 72 hours after Cre. (**D**) Bottom: Quantification of replication tract lengths of *Rtf2^−/lox^Trp53^−/−^* MEFs treated with CDK1/2i (Cdk1/2 Inhibitor III, MilliporeSigma) 72 hours after Cre. (**E**) Bottom: Integrated nuclear signal of EdU in EdU^+^ *Rtf2^−/−^* MEF clones complemented with empty vector (EV) or *Rtf2* cDNA. (**F**) Top: Nucleoside (dN) treatment and labeling scheme for DNA combing. Bottom: Quantification of replication tract length of *Rtf2^+/lox^* versus *Rtf2^−/lox^ Trp53^−/−^* MEFs treated with dNs 72 hours after Cre. (**G**) Top: CDK1/2i treatment and labeling scheme for DNA combing. Bottom: Quantification of replication tract lengths of Parental and *RNASEH2A* knockout (*RH2A KO*) HeLa cells treated with CDK1/2i (Cdk1/2 Inhibitor III). (**H**) Top: CHK1i treatment and labeling scheme for DNA combing. Bottom: Quantification of replication tract lengths of primary *Rtf2^−/lox^* MEFs 72 hours after Cre. Experiments conducted at least three times in biological replicates with consistent results for (A) to (G). Experiment conducted twice in biological replicates with consistent results for (H). Outliers removed with ROUT (1%) for [(B) to (H)]. Significance evaluated by Kruskal-Wallis ANOVA with a Dunn’s posttest for [(B) to (H)].

Cells lacking RTF2 exhibited increased levels of chromatin-bound CDC45 across S phase, further suggesting increased origin firing in these cells (Fig. 3, A and B). To investigate the relationship between origin firing and replication rates in RTF2-deficient cells, we performed DNA combing experiments upon acute exposure to inhibitors of origin firing. Acute inhibition of CDK1/2 in Cre-treated *Rtf2^−/lox^;Trp53^−/−^* MEFs rescued both excess origin firing and slow replication rates (Fig. 3, C and D). Inhibition of CDC7, another kinase required for replication initiation, yielded a dose-dependent rescue of EdU incorporation in an SV40-immortalized *Rtf2^−/−^* clone (Fig. 3E). Similarly, replication speed could be rescued in Cre-treated *p53^−/−^;Rtf2^−/lox^* MEFs by supplying cells with an excess of nucleosides (Fig. 3F). CDK1/2 inhibitor (CDK1/2i) did not restore replication rates in an RNASEH2A knockout HeLa clone, suggesting that RTF2’s participation in origin firing control, like its participation in the S-G_2_ checkpoint, is independent of its function in RNase H2 recruitment (Fig. 3G).

Given the overlapping phenotypes of RTF2- and CHK1-deficient cells, we asked whether RTF2 participates in CHK1-mediated origin firing control. In RTF2-competent cells, acute inhibition of CHK1 reduced replication tract length in a DNA fiber assay (Fig. 3H). In the setting of RTF2 loss, however, replication tract lengths were unchanged by CHK1 inhibition, suggesting that RTF2 and CHK1 function in the same pathway to restrain origin firing in S-phase cells, with their loss or inhibition leading to excessive origin firing that drives the depletion of essential replication substrates and slows replication fork progression.

### RTF2 is necessary for CHK1 localization to replication forks

**Fig. 4.**
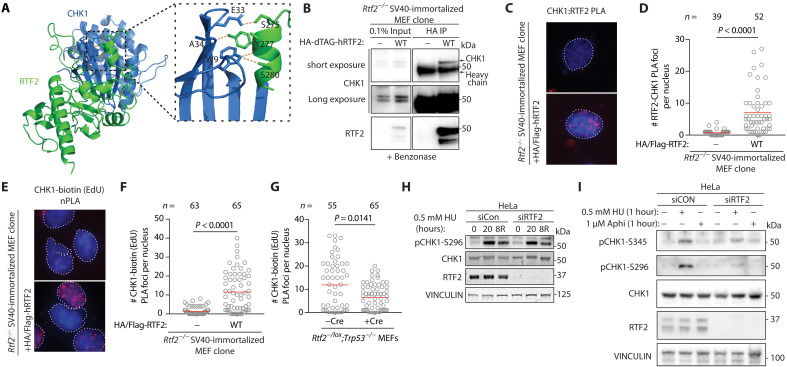
RTF2 is necessary for CHK1 localization to replication forks and activation in response to acute replication stress. (**A**) AlphaFold-Multimer prediction of RTF2-CHK1 interaction as identified in an in silico protein-protein interaction screen between RTF2 and 228 DNA damage and replication-associated proteins. Unstructured portions of RTF2 have been removed for clarity. (**B**) Representative immunoblot following immunoprecipitation of exogenously expressed HA-tagged RTF2 from an *Rtf2^−/−^* MEF clone. (**C**) Representative images of proximity ligation assay (PLA) foci in an *Rtf2^−/−^* MEF clone between exogenously expressed Flag/HA-hRTF2 and endogenous CHK1. DAPI stains the nucleus. (**D**) Quantification of RTF2-CHK1 PLA foci from experiment shown in (C). (**E**) Representative images of CHK1 nascent PLA (nPLA) in an *Rtf2*^*−/−*^ MEF clone ± exogenously expressed Flag/HA-hRTF2. DAPI stains the nucleus. (**F**) Quantification of CHK1 nPLA foci from experiment shown in (E). (**G**) Quantification of CHK1 nPLA foci in *Rtf2^−/lox^;Trp53^−/−^* MEFs 72 hours after Cre. (**H**) Representative immunoblot in HeLa cells 72 hours after transfection with indicated siRNA and treated with 0.5 mM hydroxyurea (HU) (0, untreated; 20, 20-hour treatment; 8R, 20-hour treatment followed by 8 hour release). (**I**) Representative immunoblot in HeLa cells 72 hours after transfection with indicated siRNA and treated with 0.5 mM HU or 1 μM aphidicolin (Aphi) for 1 hour. Experiments conducted at least three times in biological replicates with consistent results for (C) to (I). *n* indicates number of nuclei scored for each condition in (D), (F), and (G). Outliers removed with ROUT (1%) for (D) and (F). Significance evaluated by unpaired parametric *t* test for [(D), (F), and (H)].

To explore the mechanism of RTF2’s function in S-phase progression, we conducted an in silico screen for RTF2-interacting proteins using AlphaFold3-Multimer (*25*–*27*). Among the 228 DNA damage and replication stress response proteins surveyed, the most confident hit was CHK1 (table S1). A short α-helix near the C terminus of RTF2 was predicted to form hydrogen bonds with residues in CHK1’s N-terminal kinase domain (Fig. 4A). The high-confidence interaction was observed in three of the three AlphaFold-Multimer models surveyed during the screen (fig. S5A). We conducted additional modeling using AlphaFold-Multimer, and, again, the high-confidence interaction was detected in five of the five models (fig. S5B) (*25*).

To validate the putative interaction, we performed immunoprecipitation of exogenously tagged RTF2 and confirmed its interaction with endogenous CHK1 (Fig. 4B). In addition, the two proteins colocalized in a proximity ligation assay (PLA), which showed specific RTF2-CHK1 PLA foci formation in an SV40-immortalized *Rtf2^−/−^* MEF clone when RTF2 cDNA was expressed (Fig. 4, C and D).

CHK1 has been identified at replication forks by nascent chromatin capture and isolation of protein on nascent DNA (*28*, *29*). Given the S-G_2_ checkpoint bypass and replication phenotypes observed in RTF2-deficient cells, we asked whether CHK1 was at the replisome in RTF2-deficient cells using CHK1 nascent PLA (nPLA) to assess proximity of CHK1 to newly replicated (nascent) DNA. The amount of CHK1 at the replisome was significantly reduced in both an *Rtf2^−/−^* MEF clone and Cre-treated *Rtf2^−/lox^;Trp53^−/−^* MEFs (Fig. 4, E to G). Together, these findings suggest that RTF2 is necessary for CHK1 localization to replication forks to facilitate both its participation in the S-G_2_ checkpoint and its regulation of unperturbed DNA replication by limiting the number of actively replicating origins. Thus, RTF2 is necessary for CHK1 signaling at unperturbed replication forks.

As CHK1 also responds to replication stress to prevent cell cycle progression while DNA is damaged, we asked whether RTF2 is necessary for CHK1 activation under these circumstances (*1*). CHK1 was robustly activated, as indicated by its autophosphorylation at Ser^296^, independent of RTF2 after a 20-hour exposure to low-dose hydroxyurea (HU) (Fig. 4H) (*30*). This could be secondary to formation of DNA double-strand breaks under these conditions. However, CHK1 activation (as indicated by its autophosphorylation at Ser^296^) and ATR activation (as indicated by CHK1 phosphorylation at Ser^345^) was visibly diminished in RTF2-deficient cells following low and acute exposure to HU (Fig. 4I). We conclude that, as observed during unperturbed replication, localization of CHK1 to replication forks by RTF2 promotes CHK1 activation by ATR to facilitate the rapid response to replication stress. However, RTF2 may not be required for CHK1 activation after prolonged exposure to replication stress. We note that we cannot assess the influence of RTF2 on the basal activation of CHK1 using the pCHK1-Ser^296^ antibody as the signal induced by unperturbed replication is negligible.

**Fig. 5.**
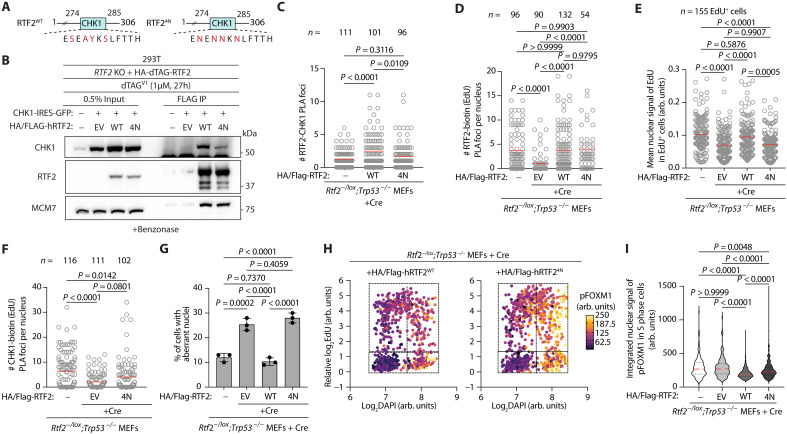
RTF2-CHK1 interaction is necessary for S-G_2_ checkpoint maintenance. (**A**) Map of CHK1-interacting domain within the C terminus of RTF2. Putative hydrogen bonding residues are indicated in red. These residues have been mutagenized to asparagine in RTF2^4N^. (**B**) Representative immunoblot following immunoprecipitation of exogenously expressed HA/Flag-RTF2 and CHK1 from RTF2 knockout (KO) 293T cells stably expressing HA-dTAG-RTF2. HA-dTAG-RTF2 was depleted for 27 hours before immunoprecipitation of FLAG-RTF2. (**C**) Quantification of RTF2-CHK1 PLA foci in *Rtf2^−/lox^;Trp53^−/−^* MEFs expressing EV, HA/Flag-RTF2^WT^, or HA/Flag-RTF2^4N^ 72 hours after Cre. (**D**) Quantification of RTF2 nPLA foci in *Rtf2^−/lox^;Trp53^−/−^* MEFs expressing EV, HA/Flag-RTF2^WT^, or HA/Flag-RTF2^4N^ 72 hours after Cre. (**E**) Mean nuclear signal of EdU in EdU^+^ *Rtf2^−/lox^;Trp53^−/−^* MEFs expressing EV, HA/Flag-RTF2^WT^, or HA/Flag-RTF2^4N^ 72 hours after Cre. One hundred fifty-five EdU^+^ nuclei were scored per condition. arb. units, arbitrary units. (**F**) Quantification of CHK1 nPLA foci in *Rtf2^−/lox^;Trp53^−/−^* MEFs expressing EV or HA/Flag-RTF2^4N^ 72 hours after Cre. (**G**) Percentage of cells with aberrant nuclei, including micronuclei, anaphase bridges, and multilobed nuclei. Each dot represents mean percentage from an independent biological replicate. (**H**) Representative pFOXM1 QIBC plots of *Rtf2^−/lox^;Trp53^−/−^* MEFs expressing HA/Flag-RTF2^WT^ or HA/Flag-RTF2^4N^ 72 hours after Cre. (**I**) Quantification of nuclear pFOXM1 staining across S phase of cells represented in (H). Experiments conducted twice in biological replicates with consistent results for (B) to (D) and (F). Experiments conducted three times in biological replicates with consistent results for (E), (H), and (I). *n* indicates number of nuclei scored for each condition in (C), (D), and (F). Outliers removed with ROUT (1%) for (I). Significance evaluated by Kruskal-Wallis ANOVA with a Dunn’s posttest for [(C) to (G) and (I)].

We next attempted to abrogate the interaction between RTF2 and CHK1. We examined the modeled interaction interface and made asparagine substitutions at the putative hydrogen bonding residues (Fig. 5A). Exogenously expressed RTF2^4N^ immunoprecipitated less CHK1 and formed fewer RTF2-CHK1 PLA foci than RTF2^WT^ in cells (Fig. 5, B and C). Despite its proper localization to replication forks by nPLA, the RTF2^4N^ mutant failed to rescue replication by EdU incorporation in Cre-treated *Rtf2^−/lox^;Trp53^−/−^* MEFs (Fig. 5, D and E). Moreover, abrogation of the RTF2-CHK1 interaction decreased recruitment of CHK1 to the replisome as assessed by nPLA (Fig. 5F). Like RTF2-deficient cells, these cells formed abnormal nuclei and accumulated pFOXM1 during S phase, indicative of S-G_2_ checkpoint bypass (Fig. 5, G to I).

Collectively, these data revealed that even partial disruption of the RTF2-CHK1 interaction leads to slow replication and S-G_2_ checkpoint bypass. We were unable to fully abrogate the interaction between RTF2 and CHK1, however, and asked whether another replication fork factor may participate in S-phase regulation of CHK1.

### CLASPIN assists in regulating fork localization of CHK1

Many reports have shown that CLASPIN, an essential replisome component and member of the fork protection complex, is necessary for proficient DNA replication and ATR-dependent CHK1 activation in the setting of replication stress (*31*–*35*). We thus hypothesized that CLASPIN may contribute to S-G_2_ checkpoint maintenance during unperturbed replication by affecting CHK1 localization to the replication fork.

**Fig. 6.**
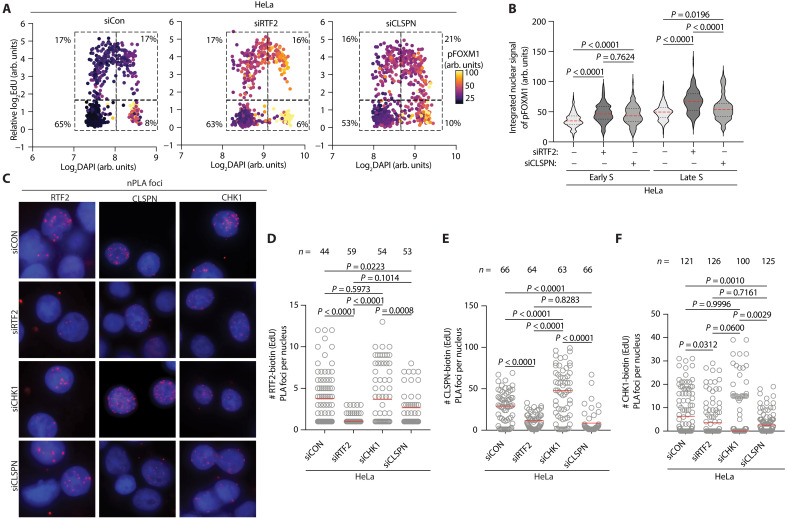
CLASPIN assists in fork localization and S-G_2_ checkpoint function of CHK1. (**A**) Representative pFOXM1 QIBC plots of HeLa cells 72 hours after transfection with indicated siRNA. (**B**) Quantification of nuclear pFOXM1 levels across early versus late S phase of cells represented in (A). arb. units, arbitrary units. (**C**) Representative images of indicated nPLA foci in HeLa cells 72 hours after transfection with indicated siRNA for experiments quantified in (D) to (F). DAPI stains the nucleus. (**D**) Quantification of RTF2 nPLA foci per nucleus in HeLa cells 72 hours after transfection with indicated siRNA. (**E**) Quantification of CLSPN nPLA foci per nucleus in HeLa cells 72 hours after transfection with indicated siRNA. (**F**) Quantification of CHK1 nPLA foci per nucleus in HeLa cells 72 hours after transfection with indicated siRNA. Experiments conducted at least three times in biological replicates with consistent results for (A) to (D) and (F), with the exception of the CLSPN nPLA (C and E), which was conducted twice in biological replicates. Significance evaluated by Kruskal-Wallis ANOVA with a Dunn’s posttest for [(B) to (F)].

CLASPIN depletion by siRNA led to increased pFOXM1 levels in replicating cells (Fig. 6, A and B; and fig. S6, A and B). This phenotype was particularly pronounced in early S phase, when CLASPIN and RTF2 depletion led to similar increases in pFOXM1 (Fig. 6B). Similar results were obtained in primary *Rtf2^−/lox^* MEFs transduced with Cre and shRNA against Claspin (fig. S6, C to E). In this experimental system, dual depletion of RTF2 and CLASPIN did not yield an additive increase in pFOXM1 accumulation, which further suggested that RTF2 and CLASPIN may function in the same checkpoint control pathway (fig. S6E).

To better understand how RTF2, CLASPIN, and CHK1 may function together to maintain the S-G_2_ checkpoint, we assessed their fork occupancy in RTF2-, CLASPIN-, and CHK1-depleted cells by nPLA. First, we confirmed that depletion of CHK1 did not cause loss of either RTF2 or CLASPIN from the replisome (Fig. 6, C to E; and fig. S6, F to I). Depletion of RTF2 or CLASPIN each led to decreased localization of CHK1 to sites of active replication (Fig. 6, C and F; and fig. S6, J and K). Thus, it appears that both RTF2 and CLASPIN function to recruit CHK1 to the replisome and prevent S-G_2_ checkpoint bypass. Moreover, depletion of RTF2 or CLASPIN led to reciprocal decrease of the other protein at nascent DNA, as visualized by nPLA (Fig. 6, C to E). Whether RTF2 and CLASPIN directly interact and whether this direct interaction is necessary for CHK1’s localization to sites of active replication needs to be determined.

## DISCUSSION

**Fig. 7.**
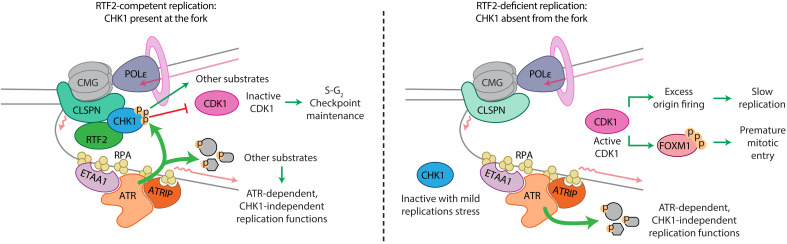
A model for RTF2’s function at the unperturbed replication fork. (**Left**) RTF2 and CLASPIN function to recruit CHK1 to progressing replication forks. ATR-ATRIP is activated when ETAA1 binds to RPA coating exposed ssDNA at the replication fork. Activated ATR then phosphorylates its downstream targets, including CHK1. Tethered by RTF2 and CLASPIN, fork-localized CHK1 suppresses CDK1 to limit origin firing at nearby dormant origins and maintain the S-G_2_ checkpoint. Of note, not all substrates of CHK1 at the fork may be ATR or RTF2 dependent and ATR has many substrates outside of CHK1. (**Right**) CHK1 is not recruited to RTF2-deficient forks, leading to heightened CDK1 activity despite active replication. CDK1 activity drives adjacent origin firing, which depletes replication substrates and activates the mitotic transcriptional program while replication proceeds. Lack of RTF2 may directly or indirectly affect CLASPIN levels at the fork (depicted as lighter color of CLASPIN in the absence of RTF2). Upon prolonged damage or other stimuli, CHK1 can be activated without being tethered to the fork by RTF2.

Here, we have described how CHK1 is localized to sites of unperturbed replication to maintain the S-G_2_ checkpoint. Until now, it has been assumed that CHK1 phosphorylation was solely dependent on ATR activation by ssDNA binding of RPA and associated proteins. However, our data show that CHK1 localization to the replisome occurs via a dedicated recruitment pathway that is dependent on RTF2 and CLASPIN. We posit that recruitment creates a localized hub of CHK1 that is poised to be activated by ATR to limit CDK1 activity, which, on one hand, promotes high replication rates by limiting origin firing and, on the other hand, prevents cell division before replication completion. Disruption of CHK1 recruitment, via loss of RTF2 or CLASPIN from the replication fork, alleviates local inhibition of CDK1. This, in turn, drives unrestrained origin firing, which depletes essential replication substrates and causes slow replication rates, and inappropriately triggers the mitotic transcriptional program despite ongoing replication (Fig. 7). Considering the essential nature of all the proteins under study, the complete genetic interdependencies are difficult to disentangle with current tools. We note that ATR has other functions that are not RTF2 or CHK1 dependent during DNA replication. In turn, RTF2 also has ATR- and CHK1-independent functions, and, upon prolonged DNA damage, CHK1 can be activated without being tethered to the fork by RTF2, potentially by ATM or DNAPK.

We find that maintenance of the S-G_2_ checkpoint also depends on CLASPIN, which has long been known to regulate activation of CHK1 by ATR during replication stress. CLASPIN was first identified as a CHK1 binding protein in *Xenopus* egg extracts, and further work in *Xenopus* and human cells showed that the CLASPIN-CHK1 interaction functioned downstream of replication stress or DNA damage signaling to amplify CHK1 activation (*33*, *35*–*38*). Studies of the CLASPIN-CHK1 interaction in the context of unperturbed replication have been limited, although CLASPIN associates with replicating chromatin and was recently shown to be an intrinsic component of the human replisome that regulates histone recycling to daughter DNA strands (*34*, *37*, *39*–*41*). CLASPIN depletion, like CHK1 or RTF2 depletion, leads to slow DNA replication and firing of excess replication origins (*31*, *32*, *42*). Whether RTF2 interacts directly with CLASPIN or whether CLASPIN functions in recruitment of RTF2 to the replication fork remains to be seen. The reciprocal effects of RTF2 and CLASPIN loss suggest some level of interdependence of these proteins, although the extent to which these effects are direct is unclear. However, our findings implicate both proteins in the regulation of CHK1 localization to the replication fork and the suppression of premature mitotic entry during ongoing replication.

We first identified RTF2 as enriched at stressed replication forks in cells depleted of the DDI1/2 proteasome shuttle proteins, indicating that DDI1/2 function to dynamically regulate RTF2’s levels at the replication fork (*19*). DDI1/2-depleted cells exhibited genome instability phenotypes, including sensitivity to replication stress, ssDNA accumulation, and inefficient replication restart after stalling, that were rescued upon depletion of RTF2, indicating that DDI1/2-dependent regulation of RTF2 at the replication fork is necessary for genome maintenance (*19*). Here, we show that RTF2 recruits CHK1 to the replisome and that this localization is necessary for control of progression through an unperturbed S phase. We find that RTF2-depleted cells fail to robustly activate CHK1 during acute, but not prolonged, replication stress. This finding suggests that localization of CHK1 to the replisome by RTF2 promotes the rapid transmission of the replication stress response, likely by decreasing the distance between ATR and CHK1. How RTF2’s removal from stressed replication forks by DDI1/2 affects the RTF2-CHK1 interaction, CHK1’s fork occupancy, and the response to replication stress remains an open question.

## MATERIALS AND METHODS

### Cell culture

MEFs, including SV40-immortalized *Rtf2^−/−^* clonal line as well as primary *Rtf2^−/lox^* and *Rtf2^−/lox^;Trp53^−/−^* lines, were generated as previously reported (*20*). For conditional *Rtf2* cell lines, experiments were performed 72 hours after transduction with pMMP Hit & Run Cre recombinase as indicated (*43*).

Primary MEFs were maintained in Dulbecco’s modified Eagle’s medium (DMEM), supplemented with 15% (v/v) fetal bovine serum (FBS), 1× nonessential amino acids, 1× GlutaMAX, and 100 U of penicillin-streptomycin/ml. Transformed MEFs, 293T cells, and HeLa cells were maintained in DMEM supplemented with 10% FBS, 1× nonessential amino acids, 1× GlutaMAX, and 100 U of penicillin-streptomycin/ml.

### Viral transductions

cDNAs were delivered by retroviral transduction after packaging in human embryonic kidney 293T cells. To package virus, 5 × 10^6^ 293T cells were plated the day before transfection. DNA and viral packaging vectors were transfected into cells with TransIT 293 transfection reagent (Mirus Bio) according to the manufacturer’s protocol. Media were changed the next day. Viral supernatant was harvested and filtered (0.45 μM) at 24, 36, 48, and 60 hours posttransfection. Target cells were infected twice, 12 hours apart, with viral supernatants supplemented with polybrene (4 μg/ml). Media were changed the following day. Two days after infection, stably expressing cells were selected with the appropriate agent [puromycin (2 μg/ml), hygromycin (100 μg/ml), blasticidin (600 μg/ml), and neomycin (600 μg/ml)]. Cell lines were validated by immunoblotting.

HeLa cells expressing HA-dTAG-RH2A were generated by lentiviral transduction of HeLa RH2A KO cell line (gifted from D. Durocher) with the lentiviral dTAG expression plasmid described below (*44*). dTAGV-1 (V-1) was used to drive the degradation of the HA-dTAG-RH2A as previously described (*23*).

293T cells expressing HA-dTAG-RTF2^PAM^ were generated by lentiviral transduction of a parental 293T cell line with the lentiviral dTAG expression plasmid described below. Knockout of endogenous RTF2 was then performed by ribonucleoprotein delivery of Cas9 complexed with the following guide RNA for RTF2: CAAGCACGATGACCTCCAG. Cells were single-cell cloned and screened for knockout of endogenous RTF2 by immunoblotting. Treatment with dTAGV-1 (V-1) was used to drive the degradation of the HA-dTAG-RTF2^PAM^ as previously described (*23*).

### siRNA transfections

Cells were forward transfected using Lipofectamine RNAiMAX Transfection Reagent according to the manufacturer’s instructions. siRNA-lipid complexes were added to the bottom of the plate, and single-suspension cells were seeded on top of the complexes. Knockdown was measured by IF or western.

### Plasmid generation and mutagenesis

RTF2 mutagenesis was performed either through site-directed mutagenesis or through gene block purchase (Twist Biosciences). Gene blocks were designed with attB overhangs and cloned into pDONR223 with BP Clonase II Enzyme Mix. Site-directed mutagenesis of pENTR-RTF2 was performed with Agilent QuikChange kits according to manufacturer’s protocol. pENTR derivatives were screened for intended mutations by Sanger sequencing (Azenta) and cloned into destination vectors with LR clonase II Enzyme Mix. Reactions were transformed into chemically competent DH5-α or Stlb3 *Escherichia coli* cells and plated onto LB/agar plates with the appropriate bacterial selection [spectinomycin (50 μg/ml) or ampicillin (100 μg/ml)]. Clones were screened by Sanger sequencing (Azenta), and correct clones were maxi-prepped for tissue culture use.

HA-dTAG-hRNASEH2A and HA-dTAG-hRTF2 were generated by LR reaction with appropriate pENTR clone and destination vector containing N-terminal FK506-Binding Protein (FKBP) sequence (pLEX_305-N-dTAG) (*23*). HA-dTAG-hRTF2^PAM^, which contained two silent mutations (c.346C>A and c.348G>A) within the PAM sequence for the guide RNA used to knockout endogenous RTF2 as described above, was generated by LR reaction with a PAM-mutated pENTR clone and destination vector containing N-terminal FKBP sequence (pLEX_305-N-dTAG) (*23*).

A gene block of the following sequence was purchased from Twist Bioscience for Gateway cloning of RTF2^4N^:

attB1_RTF2^4N^_attB2:

GGGGACAAGTTTGTACAAAAAAGCAGGCTTAATGGGTTGCGACGGGGGAACAATCCCCAAGAGGCATGAACTGGTGAAGGGGCCGAAGAAGGTTGAGAAGATCGACAAAGATGCTGAATTAGTGGCCCAATGGAACTATTGTACTCTAAGTCAGGAAATATTAAGACGACCAATAGTTGCCTGTGAACTTGGCAGACTTTATAACAAAGATGCCGTCATTGAATTTCTCTTGGACAAATCTGCAGAAAAGGCTCTTGGGAAGGCAGCATCTCACATTAAAAGCATTAAGAATGTGACAGAGCTGAAGCTTTCTGATAATCCTGCCTGGGAAGGGGATAAAGGAAACACTAAAGGTGACAAGCACGATGACCTCCAGCGGGCGCGTTTCATCTGCCCCGTTGTGGGCCTGGAGATGAACGGCCGACACAGGTTCTGCTTCCTTCGGTGCTGCGGCTGTGTGTTTTCTGAGCGAGCCTTGAAAGAGATAAAAGCGGAAGTTTGCCACACGTGTGGGGCTGCCTTCCAGGAGGATGATGTCATCATGCTCAATGGCACCAAGGAGGATGTGGACGTGCTGAAGACAAGGATGGAGGAGAGAAGGCTGAGAGCGAAGCTGGAAAAGAAAACAAAGAAACCCAAGGCAGCAGAGTCTGTTTCAAAACCAGATGTCAGTGAAGAAGCCCCAGGGCCATCAAAAGTTAAGACAGGGAAGCCTGAAGAAGCCAGCCTTGATTCTAGAGAGAAGAAAACCAACTTGGCTCCCAAAAGCACAGCAATGAATGAGAGCTCTTCTGGAAAAGCTGGGAAGCCTCCGTGTGGAGCCACAAAGAGGTCCATCGCTGACAGTGAAGAAAACGAGAACAACAAGAACCTCTTTACCACTCACAGCTCCGCCAAGCGCTCCAAGGAGGAGTCTGCCCACTGGGTCACCCACACGTCCTACTGCTTCTGAAACCCAGCTTTCTTGTACAAAGTGGTCCCC

pMD2.G was a gift from D. Trono (Addgene, plasmid no. 12259; http://n2t.net/addgene:12259; RRID:Addgene_12259). psPAX2 was a gift from D. Trono (Addgene, plasmid no. 12260; http://n2t.net/addgene:12260; RRID:Addgene_12260). pMMP Hit & Run Cre was a gift from D. Livingston (*43*). pLEX_305-N-dTAG was a gift from James Bradner & Behnam Nabet (Addgene, plasmid no. 91797; http://n2t.net/addgene:91797; RRID:Addgene_91797) (*23*).

### Immunofluorescence and QIBC

Cells were pulsed with 10 μM EdU for 10 min, rinsed three times with phosphate-buffered saline (PBS), and fixed in 3.7% (v/v) formaldehyde in PBS at room temperature (RT) for 10 min. Cells were washed and permeabilized in 0.5% (v/v) Triton X-100 in PBS. For chromatin-bound CDC25, cells were rinsed three times with PBS and then preextracted in ice cold 0.2% Triton-X-100 in PBS on ice for 4 min before fixation. Cells were blocked in either 5% (v/v) FBS in PBS or 3% bovine serum albumin (w/v) in PBS at RT for 30 min. EdU was stained with the Click-iT EdU Alexa Fluor 488 Imaging Kit (Invitrogen, C10337) according to the manufacturer’s protocol. Primary antibodies were diluted 1:1000 in blocking buffer and incubated overnight at 4°C. Cells were washed three times for 5 min with PBS and incubated in corresponding secondary antibodies (1:1000) for 1 hour at RT. Cells were washed three times for 5 min with PBS and stained with 4′,6-diamidino-2-phenylindole (DAPI; 5 μg/ml) or Hoescht 33342 (1:10,000) for 15 min at RT. Cells were washed three times for 5 min with PBS, and coverslips were mounted on glass slides in Fluoromount-G (SouthernBiotech).

Panels of images were acquired on an Inverted Olympus IX-71 DeltaVision Image Restoration Microscope (Applied Precision) using a 20× objective. CellProfiler was used to generate a nuclear mask based on the DAPI channel. The mean or integrated nuclear signals of stained proteins were then measured within the nuclear mask. Output data from CellProfiler was plotted in GraphPad Prism to generate multiple variable plots, with integrated DAPI signal on the *x* axis and EdU incorporation on the *y* axis and color range determined by pFOXM1 or cyclin B1 integrated nuclear stain.

### Coimmunoprecipitation

Stably transduced *Rtf2^−/−^* MEFs expressing HA/Flag-RTF2 [wild type (WT) or mutant] cDNA or stably transduced 293Ts expressing HA/Flag-RTF2 (WT or mutant) cDNA and transiently expressing CHK1 were seeded the day before harvest at a concentration of 10 × 10^6^ to 20 × 10^6^ cells per 15 cm. For the RTF2^4N^ immunoprecipitation experiments, the HA-dTAG-RTF2 cell line with endogenous RTF2 knockout (described above) was used. These cells were transiently transfected with HA/Flag-RTF2 (WT or mutant) and CHK1 cDNA. The next day, dTAG^V1^ was added to the medium for 24 to 27 hours. Cells were harvested for immunuoprecipitation studies 48 hours posttransfection as described below. Cells were washed one time with cold PBS, harvested by scraping, and spun at 500*g* for 10 min at 4°C. Cells were lysed in 1 ml of cold lysis buffer [50 mM Hepes (pH 7.5), 150 mM NaCl, 2 mM MgCl2, 0.1% Tween-20, 1× Phosphatase inhibitor cocktail II, 1× protease inhibitor (Roche, cOmplete EDTA-free, 11697498001), and *N*-ethylmaleimide (2 mg/ml)]. Cells were sonicated (four times, 15A, for 10 to 15 s) and treated with benzonase for 30 min while rotating at 4°C. Lysates were centrifuged at 15,000*g* for 10 min to remove debris. Input was collected. The supernatant was incubated with anti-FLAG M2 magnetic beads (Sigma-Aldrich). The next day, samples were washed four to six times for 5 min in lysis buffer. Samples were heated for 10 min in 2× NuPAGE LDS buffer (Thermo Fisher Scientific) and dithiothreitol to elute and run on 4 to 12% bis-tris SDS–polyacrylamide gel electrophoresis (PAGE) gel. Membranes were subject to standard immunoblotting detection methods.

### Immunoblotting

Whole-cell lysates were prepared by lysing cells in clear Laemmli buffer [4% SDS, 20% glycerol, and 0.125 M tris-HCl (pH 6.8)], and protein concentration was determined using the detergent compatible (DC) protein assay (Bio-Rad) before addition of 2-mercaptoethanol and bromophenol blue. DNA was sheared by sonication, and samples were heated at 95°C for 10 min. Proteins were separated by SDS-PAGE on precast 4 to 12% bis-tris gels. Membranes were blocked for 1 hour in 5% milk in TBST [10 mM tris-HCl (pH 7.5), 150 mM NaCl, and 0.1% Tween 20] and incubated in primary antibodies for 1 to 2 hours at RT or overnight at 4°C. Membranes were washed in TBST (three times for 10 min) then incubated with horseradish peroxidase–conjugated secondary antibodies for 1 hour at RT. Membranes were washed in TBST (three times for 10 min) and detected by enhanced chemiluminescence using an Azure c300 imaging system. All antibodies are included in table S2.

### Proximity ligation assays

Cells were grown in glass-bottom 96-well plates (CellVis). For nPLA experiments, cells were labeled with 10 μM EdU for 3 min and 30 s, as determined in our previous report (*14*). Cells were rinsed three times with PBS and preextracted with ice cold 0.5% Triton X-100 in PBS on ice for 10 min, then fixed with 3% formaldehyde/2% sucrose in PBS, and blocked with DuoLink Blocking Reagent for 1 hour at RT. For nPLA experiments, EdU was then biotin clicked. Antibody (50 μl) diluted 1:1000 in DuoLink Antibody Dilution Solution was added to each well and incubated at 4°C overnight. Cells were washed three times with PBS and incubated in antibody-coupled sense and anti-sense probes to detect the light chains of rabbit and mouse immunoglobulin G, respectively, and the PLA reaction was conducted using DuoLink (Sigma-Aldrich) according to the manufacturer’s protocol.

Slides were imaged on an Axio Observer.A1 fluorescence microscope (Carl Zeiss), equipped with a Plan Apochromat 63× NA-1.4 oil objective, the AxioCam CCD camera, and the AxioVision Rel version 4.7 software. Foci were either counted manually or using CellProfiler with DAPI staining for a nuclear mask.

### DNA combing assays

Exponentially growing cells were labeled with 5-Iodo-2′-deoxyuridine (IdU) and 5-Chloro-2′-deoxyuridine (CldU) at 100 μM, according to treatment schematics included in figures. Cells were washed three times with warm PBS between label pulses. After labeling, cells were collected and washed in cold PBS and resuspended in 45 μl of Resuspension Buffer (PBS) with 0.2% NaN_3_. An equal volume of 2% molecular biology grade low melting agarose (Bio-Rad) melted in resuspension buffer was equilibrated at 55°C and added to cells to make agarose plugs. Once set, agarose plugs were ejected into digestion buffer [proteinase K (1 mg/ml), 1% *N*-lauroylsarcosine, 0.2% sodium deoxycholate, 100 mM EDTA, and 10 mM tris-HCl (pH 7.5)] and incubated overnight at 55°C. Plugs were then washed three times for 1 hour each in 1× Tris-EDTA (TE) (pH 8.5) with 100 mM NaCl before melting in 1 ml of combing buffer at 68°C for 20 min. After melting, tubes were cooled at 42°C for 10 min, and 1 μl of β-agarase (New England Biolabs) was added overnight at 42°C.

DNA was poured into Disposable DNA Reservoirs (Genomic Vision) and combed onto silanized coverslips [made in-house, as previously reported (*14*)] using the Molecular Combing System (Genomic Vision). Slides were dried for 2 hours at 65°C. Slides were denatured with 0.5 M NaOH and 1 M NaCl for 8 min, followed by dehydration in 70, 90, and 100% ethanol. Slides were blocked for 1 hour in 5% FBS in PBS. Slides were incubated with primary antibodies overnight at 4°C or 2 hours at RT, washed, and incubated with secondary antibody for 1 hour at RT. Slides were mounted with Fluoromount-G (SouthernBiotech), and fibers were imaged [Inverted Olympus IX-71 DeltaVision Image Restoration Microscope (Applied Precision)]. Fiber length was measured using Fiji software.

### DNA fiber assays

IdU and CldU were incorporated, and cell pellets were washed and collected for DNA fiber experiments as for DNA combing experiments. For DNA fibers, cells were resuspended at a concentration of 1 × 10^6^ cells/ml in cold PBS. On a clean glass coverslip, 10-μl droplets of spreading buffer (0.5% SDS, 200 mM tris-HCl at pH 7.4, and 50 mM EDTA at pH 8) were added to the top corner of a glass coverslip. Cell suspension (2.5 μl) was carefully mixed into the spreading buffer. Coverslips were incubated horizontally for 9 min at RT and then gently tilted to cause the buffer and cell suspension to run down the coverslip. Coverslips were heated for 30 min at 65°C and then fixed in methanol/acetic acid 3:1 overnight at 4°C. Coverslips were washed three times with PBS and denatured in 2.5 M HCl for 1 hour. Following three 5-min washes with PBS, coverslips were blocked in 5% FBS in PBS for 30 min at RT.

To stain, coverslips were incubated with mouse anti–5-bromo-2′-deoxyuridine (BrdU) antibody at 1:20 dilution in blocking buffer to detect IdU and rat anti-BrdU antibody at 1:40 dilution in blocking buffer to detect CldU overnight at 4°C. The next day, coverslips were washed three times with PBS, followed by five times with PBS with 0.2% Tween, and then blocked for 30 min in 5% FBS in PBS. Coverslips were incubated with secondary (Alexa Fluor) anti-rat (594) and anti-mouse (488) at a dilution of 1:300 for 1 hour at RT. Coverslips were washed three times for 5 min with 0.2% Tween in PBS, rinsed with water, and air-dried before mounting on glass slides using Fluoromount-G (SouthernBiotech). Fibers were imaged using an inverted Olympus IX-71 DeltaVision Image Restoration Microscope (Applied Precision), and the length was measured using Fiji software. Fibers (≥200) were analyzed for each experiment.

### Growth assays

For growth assays, 2 × 10^4^ cells were plated in triplicate in six-well plates. Cells were counted on subsequent days using a Z2 Coulter Counter Analyzer (Beckman Coulter). Population doublings were calculated according to the following formula: 3.32 × [log(the number of cells harvested) − log(the initial number of cells plated)].

### Quantification and statistical analysis

Significance was evaluated either by Kruskal-Wallis analysis of variance (ANOVA) with a Dunn’s posttest or by unpaired parametric *t* test using GraphPad Prism software. All *P* values are shown in the figure panels, and which test was used is indicated in the figure legend. CellProfiler and Fiji software were used for image quantification (*45*, *46*). For DNA combing assays and quantifications reporting average pFOXM1 intensity in S-phase cells, outliers were identified and removed with robust regression and OUTlier removal (ROUT) (1%). Descriptions of the statistical analyses presented in the figures can be found in the corresponding figure legends.
