# Supplementary material for: RTF2 and CLASPIN localize CHK1 to the replication fork to control S-phase progression
Source: Sci Adv. 2026 Aug 5;12(32):eaec3174. doi: 10.1126/sciadv.aec3174 (PMC13440383; doi:10.1126/sciadv.aec3174)
Supplement: Supplementary file 1 [file sciadv.aec3174_sm.pdf]

Science Advances
AAAS

Supplementary Materials for

RTF2 and CLASPIN localize CHK1 to the replication fork to control S-phase progression

Cayla Broton *et al.*

Corresponding author: Agata Smogorzewska, asmogorzewska@rockefeller.edu

Sci. Adv. **12**, eaec3174 (2026)
DOI: 10.1126/sciadv.aec3174

This PDF file includes:

Figs. S1 to S6
Tables S1 and S2

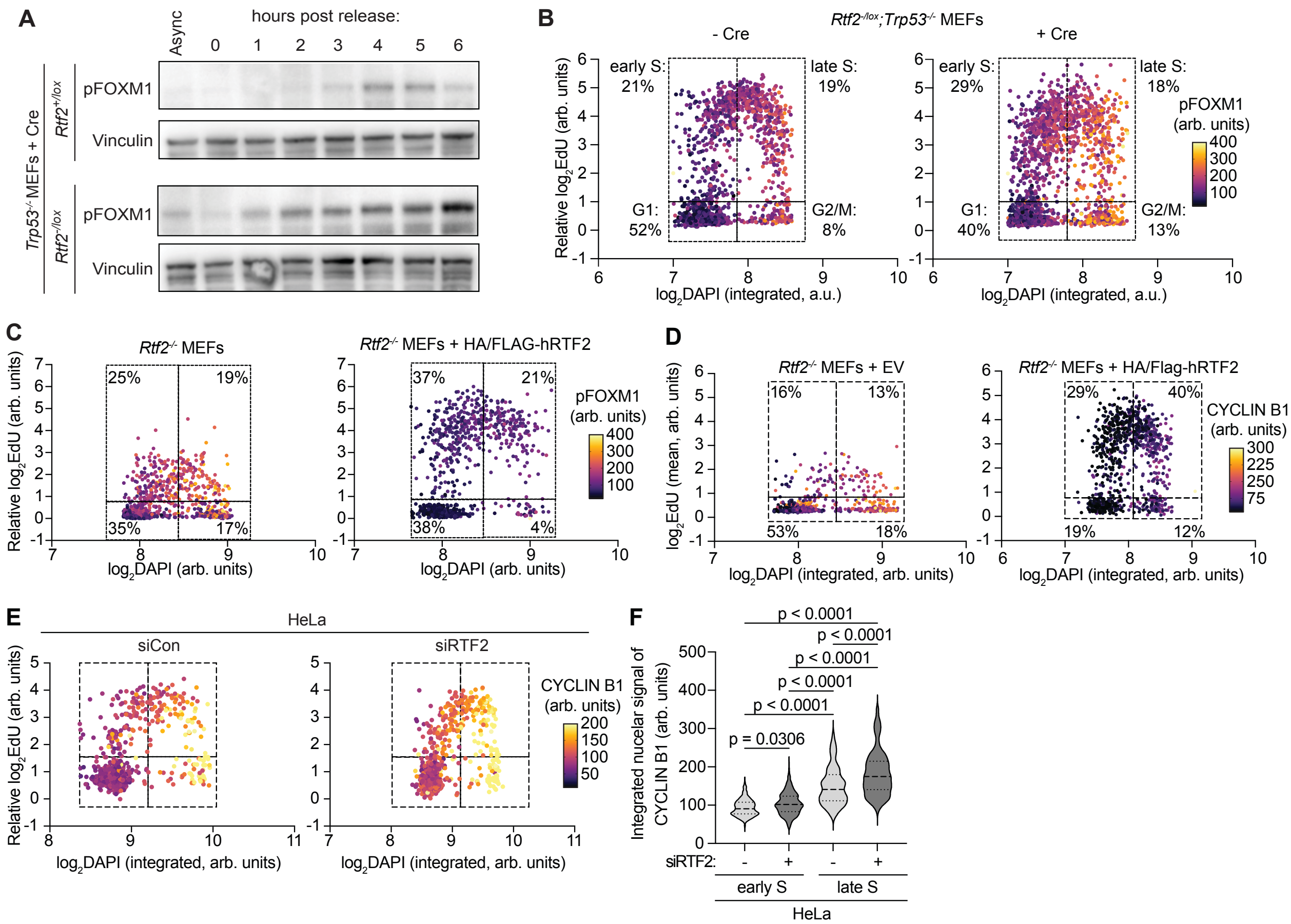

Fig. S1. RTF2-deficient cells accumulate pFOXM1 and CYCLIN B1 during S phase.

(A) Representative immunoblot of *Rtf2*$^{+/lox}$ and *Rtf2*$^{-/lox}$ *Trp53*$^{-/-}$ MEFs 72 hrs after Cre that were synchronized by single thymidine block and harvested every hour after release.

(B) pFOXM1 QIBC plots of *Rtf2*$^{-/lox}$;*Trp53*$^{-/-}$ MEFs 72 hrs after Cre. Corresponding quantification of S phase nuclear pFOXM1 is shown in Figure 1D.

(C) pFOXM1 QIBC plots of *Rtf2*$^{-/-}$MEF clone +/- HA/Flag-hRTF2. Corresponding quantification of S phase nuclear pFOXM1 is shown in Figure 1E.

(D) CYCLIN B1 QIBC plots of *Rtf2*$^{-/-}$MEF clone +/- HA/Flag-hRTF2.

(E) CYCLIN B1 QIBC plots of HeLa cells 72 hrs after transfection with indicated siRNAs.

(F) Quantification of nuclear CYCLIN B1 levels across early versus late S phase of cells shown in (E).

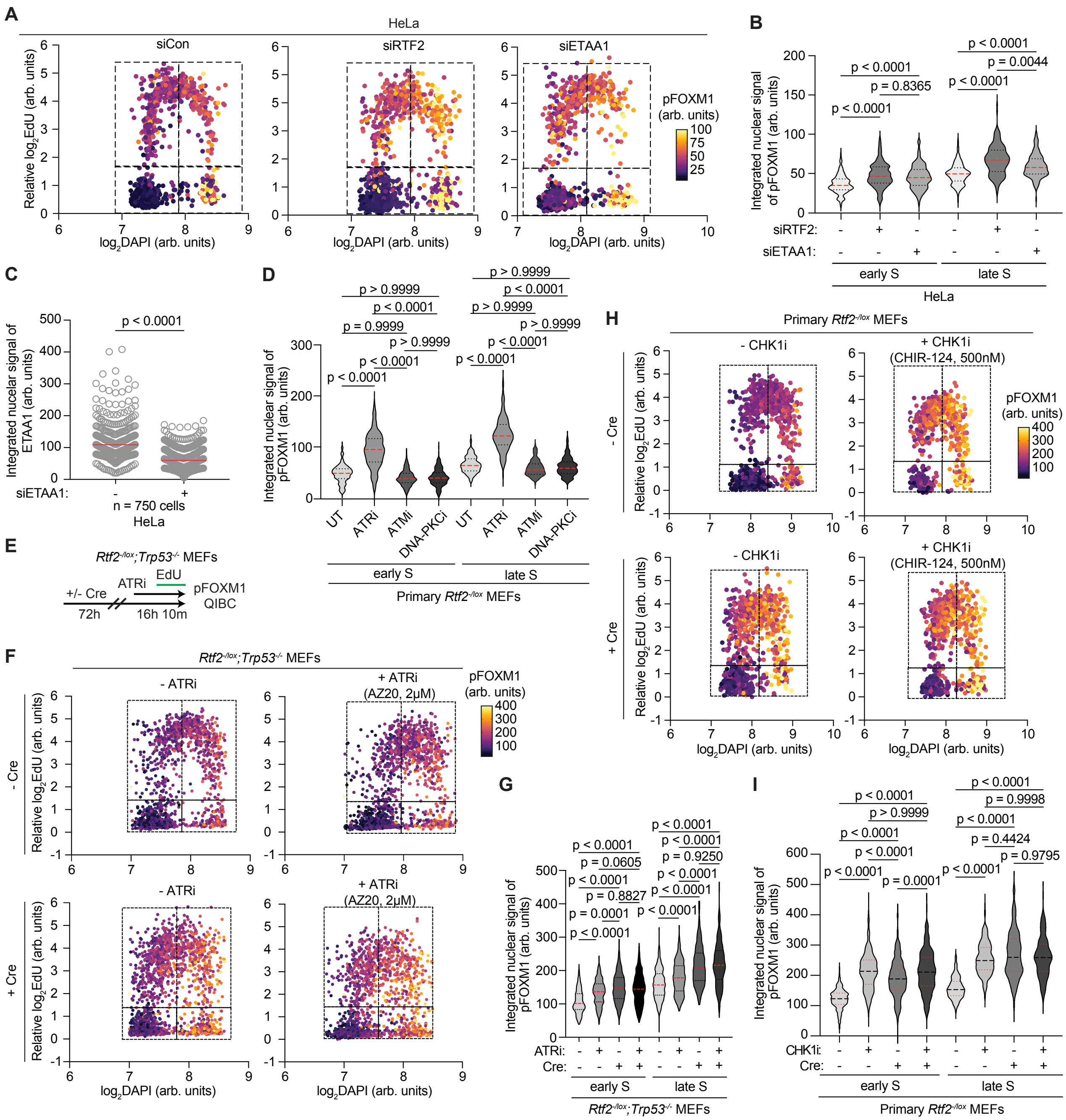
A
HeLa
siCon
siRTF2
siETAA1
Relative log2EdU (arb. units)
log2DAPI (arb. units)
pFOXM1 (arb. units)
B
Integrated nuclear signal of pFOXM1 (arb. units)
siRTF2:
siETAA1:
early S
late S
HeLa
C
Integrated nucelar signal of ETAA1 (arb. units)
siETAA1:
n = 750 cells
HeLa
D
Integrated nuclear signal of pFOXM1 (arb. units)
UT
ATRi
ATMi
DNA-PKCi
early S
late S
Primary Rtf2-/lox MEFs
E
Rtf2-/lox;Trp53-/- MEFs
+/- Cre
72h
ATRi
EdU
16h 10m
pFOXM1
QIBC
F
Rtf2-/lox;Trp53-/- MEFs
- ATRi
+ ATRi (AZ20, 2µM)
- Cre
+ Cre
G
ATRi:
Cre:
early S
late S
Rtf2-/lox;Trp53-/- MEFs
H
Primary Rtf2-/lox MEFs
- CHK1i
+ CHK1i (CHIR-124, 500nM)
I
CHK1i:
Cre:
early S
late S
Primary Rtf2-/lox MEFs

Fig. S2. RTF2-deficient cells bypass the ETAA1- and ATR-dependent intrinsic S/G2 checkpoint.

(A) pFOXM1 QIBC plots of HeLa cells 72 hrs after transfection with indicated siRNAs.

(B) Quantification of nuclear pFOXM1 levels across early versus late S phase of cells represented in (A).

(C) Integrated nuclear signal of ETAA1 in HeLa cells 72 hrs after transfection with indicated siRNA. 750 nuclei scored per condition.

(D) Quantification of nuclear pFOXM1 levels across early versus late S phase of *Rtf2$^{-/lox}$;Trp53$^{-/-}$* MEFs treated with indicated concentrations of inhibitor (ATMi (KU-55933), DNA-PKci (AZD7648), ATRi (AZ20)) for 1 hr prior to fixation. ATRi serves as positive control.

(E) ATRi treatment scheme for QIBC experiment shown in (F).

(F) pFOXM1 QIBC plots of *Rtf2$^{-/lox}$;Trp53$^{-/-}$* MEFs 72 hrs after Cre treated with ATRi as indicated.

(G) Quantification of nuclear pFOXM1 levels across early versus late S phase of cells shown in (F).

(H) pFOXM1 QIBC plots of primary *Rtf2$^{-/lox}$* MEFs 72 hrs after Cre treated with CHK1i as indicated.

(I) Quantification of nuclear pFOXM1 levels across early versus late S phase of cells shown in (H).

Experiments conducted at least three times in biological replicates with consistent results for A G. Experiment conducted one time in technical replicates with consistent results for H, I.

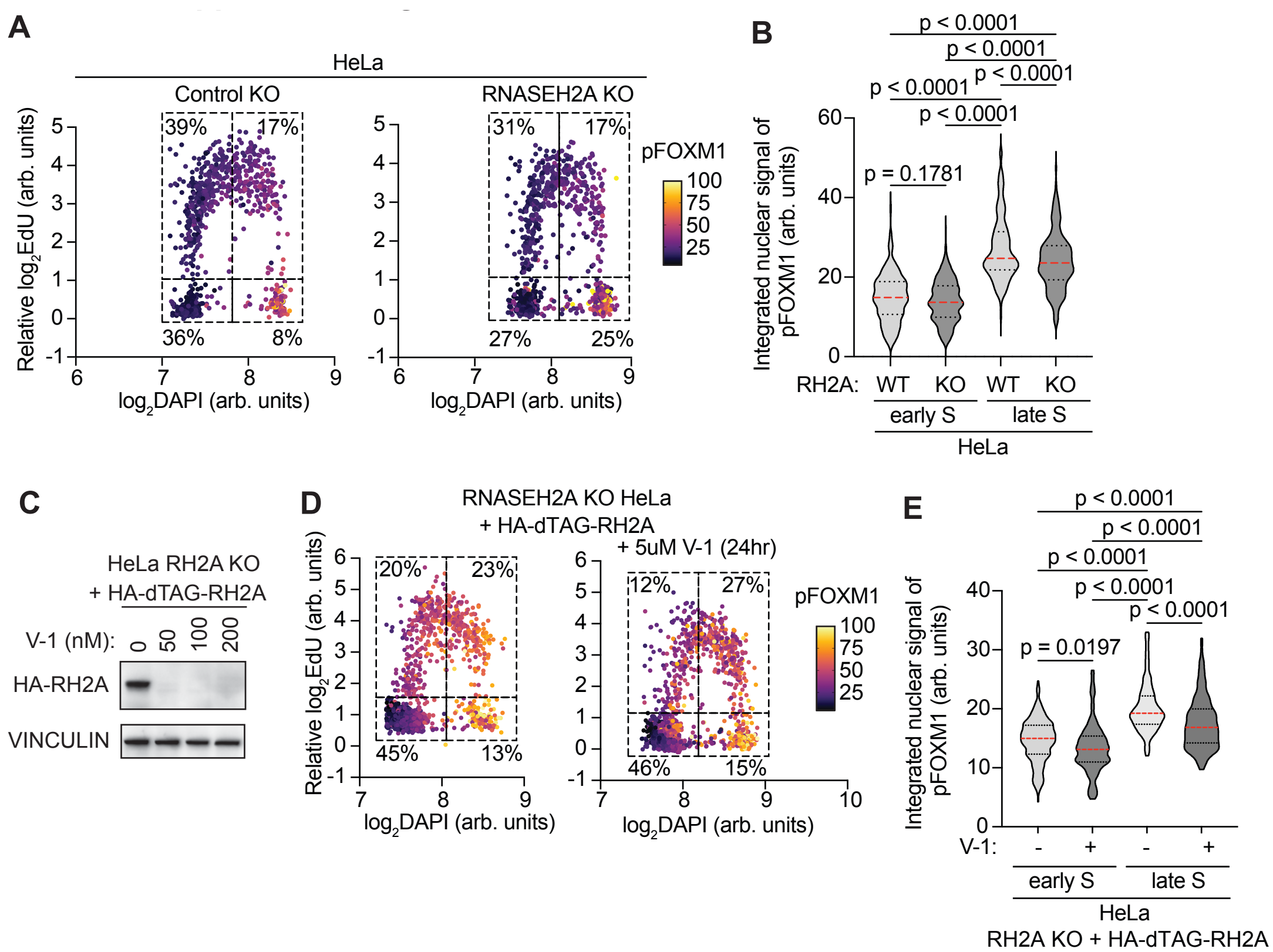

Fig. S3. Loss of RNase H2A does not cause S/G2 checkpoint bypass.

(A) pFOXM1 QIBC plots of HeLa RH2A KO versus WT cells.

(B) Quantification of nuclear pFOXM1 levels in HeLa RH2A KO versus WT cells in early versus late S phase from experiment shown in (A).

(C) Representative immunoblot of exogenously expressed HA-dTAG-RH2A in HeLa RH2A KO cells exposed to increasing amounts of $dTAG^{V}$-1 (V-1), which induces HA-dTAG-RH2A degradation.

(D) pFOXM1 QIBC plots of HeLa RH2A KO complemented with HA-dTAG-RH2A treated with increasing amounts of V-1 for 24 hours prior to fixation.

(E) Quantification of nuclear pFOXM1 levels in early versus late S phase from experiment shown in (D).

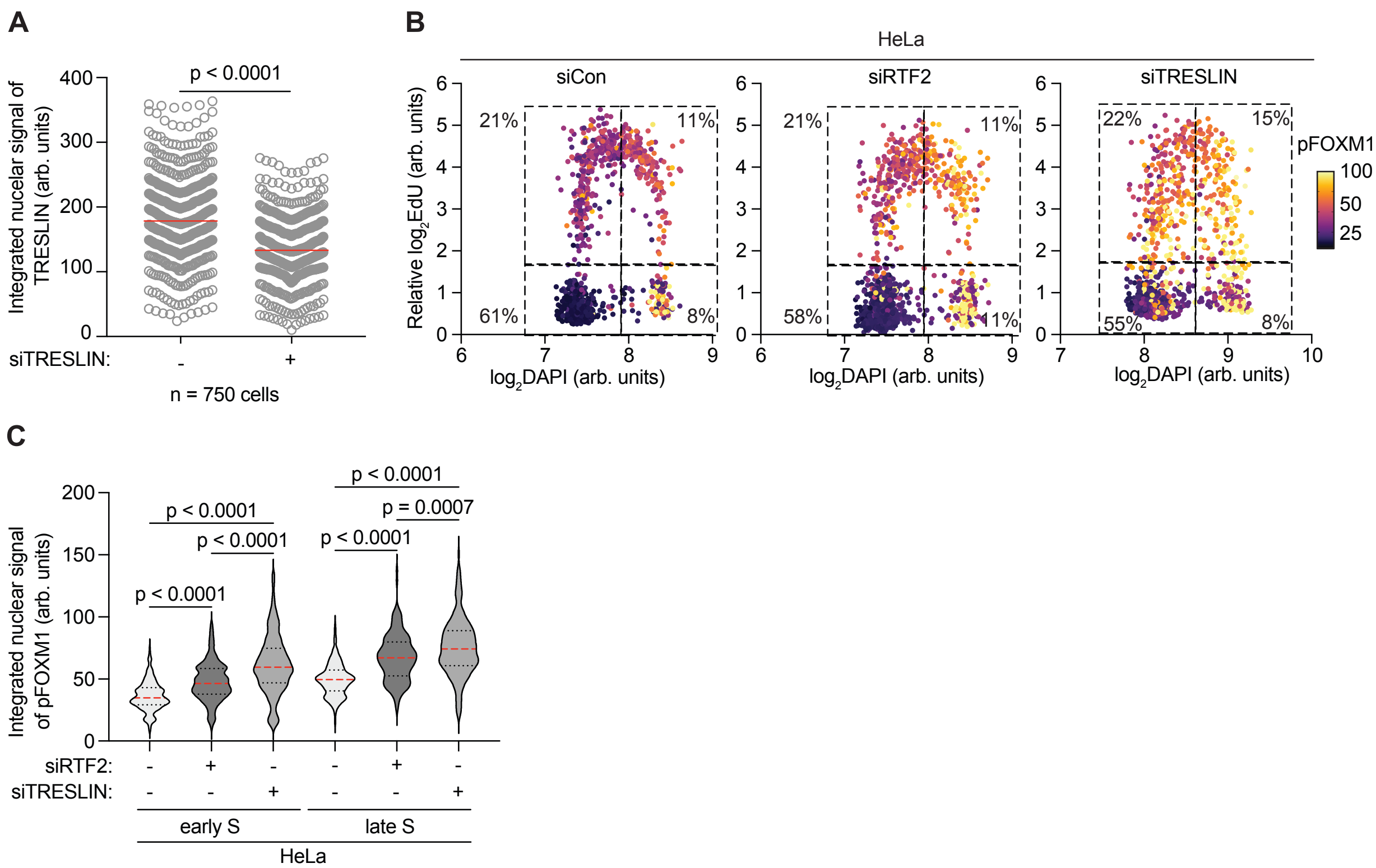

Fig. S4. RTF2- and TRESLIN-depleted cells exhibit distinct phenotypes of S/G2 checkpoint bypass.

(A) Integrated nuclear signal of TRESLIN in HeLa cells 72 hrs after transfection with indicated siRNA. 750 nuclei scored per condition.

(B) pFOXM1 QIBC plots of HeLa cells 72 hrs after transfection with indicated siRNAs.

(C) Quantification of nuclear pFOXM1 levels across early versus late S phase of HeLa cells 72 hrs after transfection with indicated siRNAs from experiment shown in (B).

Experiments conducted at least three times in biological replicates with consistent results for A-C. Significance evaluated by unpaired parametric t test for A. Significance evaluated by Kruskal-Wallis ANOVA with a Dunn's post-test for C.

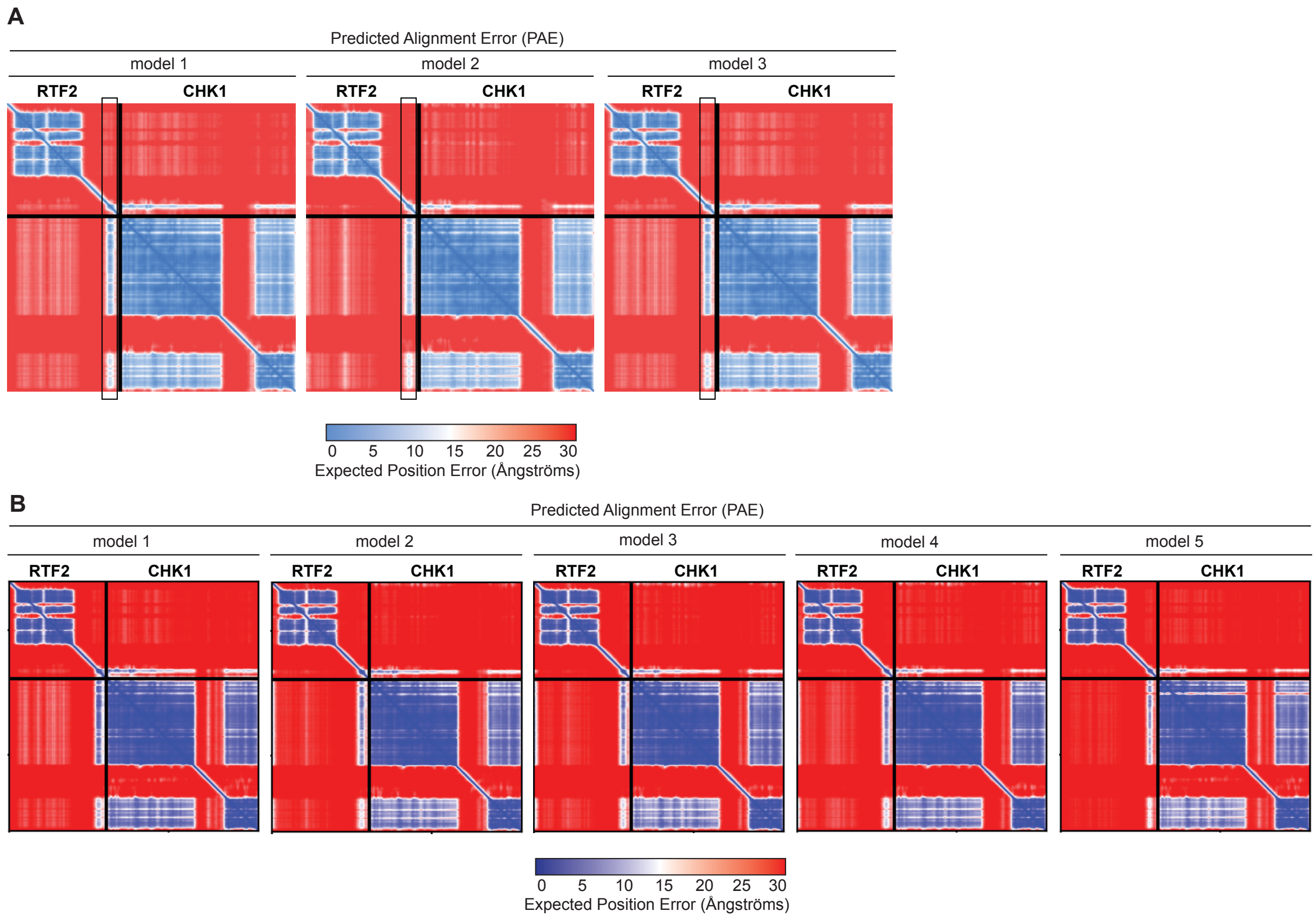

Fig. S5. Predicted alignment error (PAE) plots for RTF2 and CHK1 interaction models. PAE plots generated by the 3 AlphaFold-Multimer models in an initial *in silico* RTF2 interaction screen (A) and using AlphaFold-Multimer to generate five additional models (B). Black box indicates putative interaction identified across all models.

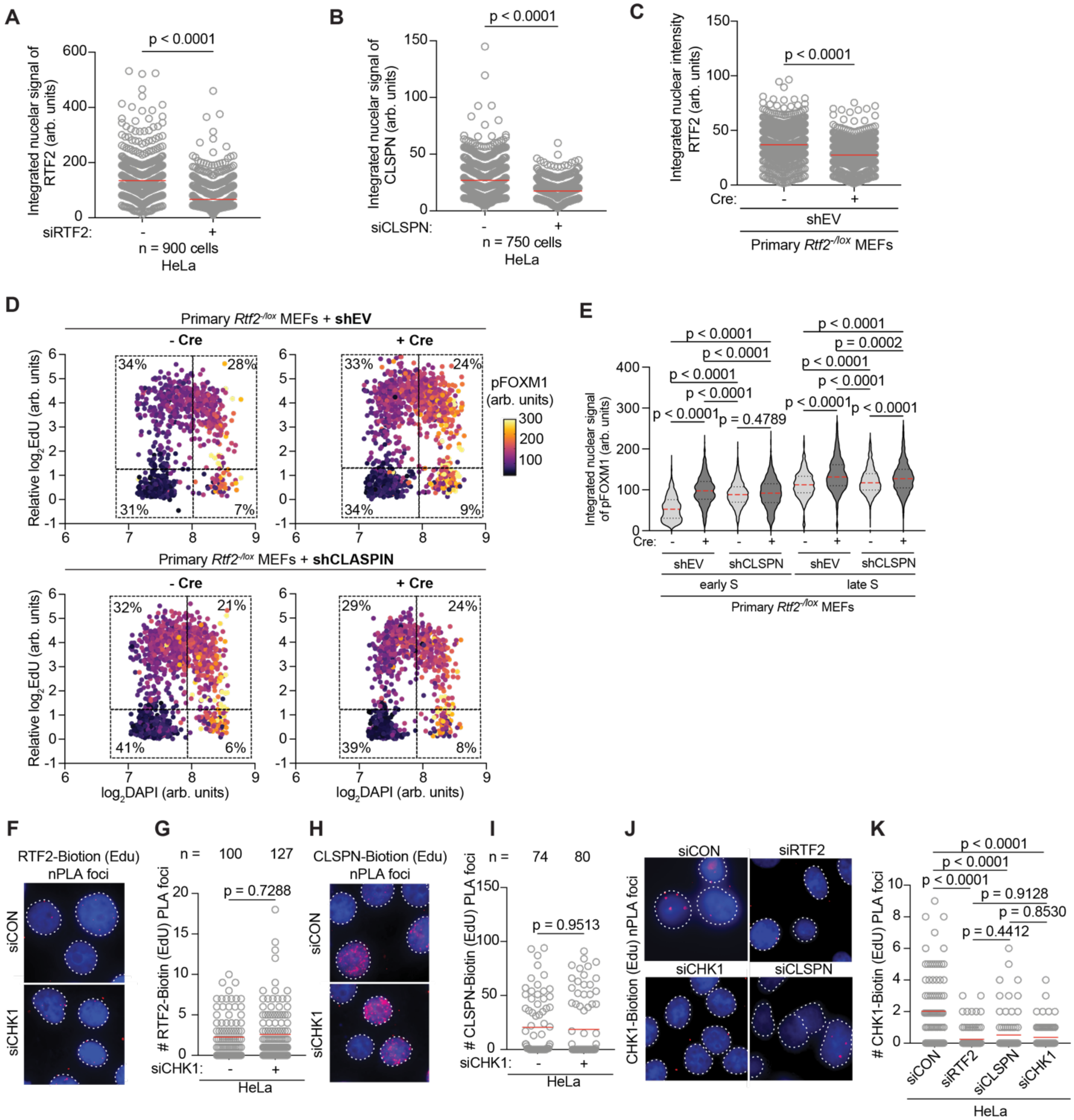

Fig. S6. RTF2 and CLASPIN regulate fork localization of RTF2-CHK1-CLASPIN complex and S/G2 checkpoint maintenance.

(A, B) Integrated nuclear signal of indicated protein in HeLa cells 72 hrs after transfection with indicated siRNA. 900 (A) or 750 (B) nuclei scored per condition.

(C) Integrated nuclear signal of RTF2 in Primary *Rtf2*$^{-/lox}$ MEFs 72 hrs after Cre for experiment shown in (D).

(D) Representative pFOXM1 QIBC plots of *Rtf2*$^{-/lox}$;*Trp53*$^{-/-}$ MEFs 72 hrs after Cre and/or shCLASPIN.

(E) Quantification of nuclear pFOXM1 levels in early versus late S phase from experiment shown in (D).
(**F, G**) Representative images (F) and quantification (G) of RTF2 nPLA foci per nucleus in HeLa cells 72 hours after transfection with indicated siRNA.
(H, I) Representative images (H) and quantification (I) of CLSPN nPLA foci per nucleus in HeLa cells 72 hours after transfection with indicated siRNA.
(**J, K**) Representative images (J) and quantification (K) of CHK1 nPLA foci per nucleus in HeLa cells 72 hours after transfection with indicated siRNA. Experiments conducted at least three times in biological replicates with consistent results for A to G, J, K. Experiments conducted twice in biological replicates with consistent results for H, I. Significance evaluated by Kruskal-Wallis ANOVA with a Dunn's post-test for E, K. Significance evaluated by unpaired parametric t test for A-C, G, I. Significance evaluated by Kruskal-Wallis ANOVA with a Dunn's post-test for E, G-I.

Table S1. ***In silico*** **screening results for RTF2 interactors in H. sapiens.** RTF2 was folded with ~230 genome maintenance proteins. The results are tabulated, with the 5 metrics of confidence described below. Results were sorted according to the average number of models in which the interaction was observed. CHK1 was the strongest predicted interactor.

**Legend**	
**Column**	**Description**
**Average # Models**	Protein pairs were folded using the three AF "models", and the average number of models that predicted the same interface was quantified.
**Max # Models**	The maximum number of models describes how many models satisfy at least some of the contacts in a predicted PPI. It is therefore equal to or greater than the average number of models value.
**Average pLDDT**	The predicted local distance difference test (pLDDT) describes the confidence that an amino acid is positioned correctly relative to neighboring residues. pLDDT values range from 0 (worst) to 100 (best). The average pLDDT of all the residues at the PPI interface in the best model is shown. Confident structure predictions generally have values >70.
**pDOCKQ**	pDOCKQ is a metric of prediction accuracy ranging from 0 to 1 (best) that takes into account the average pLDDT of all interacting residues and the number of interacting residues. High confidence predictions generally have a value > 0.25.
**Average PAE**	The predicted alignment error (PAE, measured in angstroms) is a global measure of the predicted error in the relative positioning of two residues in a structure, ranging from 0 (best) to 30 Angstroms (worst). The average PAE value for all pairs of interface residues for the highest confidence model is shown.

Protein pair	Average # Models	Max # Models	Average pLDDT	pDOCKQ	Average PAE
RTF2_HUMAN__CHK1_HUMAN__782aa	2.4	3	73.8	0.454	5.5
RTF2_HUMAN__TERF2_HUMAN__848aa	2.3	3	80.9	0.612	2.8
RTF2_HUMAN__ORC2_HUMAN__883aa	1.9	2	66.8	0.131	6.5
RTF2_HUMAN__SSRP1_HUMAN__1015aa	1.8	3	73.4	0.594	6.8
RTF2_HUMAN__FANCL_HUMAN__681aa	1.7	2	70.8	0.101	12.4
RTF2_HUMAN__TINF2_HUMAN__757aa	1.6	2	77.3	0.494	4.6
RTF2_HUMAN__SCC4_HUMAN__919aa	1.6	2	72	0.25	4.9
RTF2_HUMAN__TOP1_HUMAN__1071aa	1.6	3	71.7	0.06	14.3
RTF2_HUMAN__UBE2T_HUMAN__503aa	1.5	2	86.2	0.611	4.2
RTF2_HUMAN__RMI1_HUMAN__931aa	1.5	2	89	0.638	2.2

RTF2_HUMAN__SMAL1_HUMAN__1260aa	1.4	2	66.5	0.403	8.4
RTF2_HUMAN__EME1_HUMAN__876aa	1.3	2	68	0.316	8.9
RTF2_HUMAN__TONSL_HUMAN__1684aa	1.2	2	67.8	0.375	13.4
RTF2_HUMAN__CCNA2_HUMAN__738aa	1.1	2	73.1	0.099	11.9
RTF2_HUMAN__NSE3_HUMAN__610aa	1.1	2	68.4	0.287	7.3
RTF2_HUMAN__RMI2_HUMAN__453aa	1.1	2	75.4	0.39	14.1
RTF2_HUMAN__ABRX1_HUMAN__715aa	1.1	2	81.4	0.385	11.9
RTF2_HUMAN__PRI1_HUMAN__726aa	1.1	2	70.1	0.068	13.4
RTF2_HUMAN__ETAA1_HUMAN__1232aa	1	1	60.3	0.021	13.3
RTF2_HUMAN__BRCA1_HUMAN__2169aa	1	1	62.3	0.019	15
RTF2_HUMAN__SHLD3_HUMAN__556aa	1	1	82.8	0.617	2.8
RTF2_HUMAN__RFC3_HUMAN__662aa	1	1	83	0.018	13
RTF2_HUMAN__WAPL_HUMAN__1496aa	1	1	64	0.114	11.8
RTF2_HUMAN__ELOB_HUMAN__424aa	1	1	70.7	0.236	14.2
RTF2_HUMAN__RHNO1_HUMAN__544aa	1	1	70.8	0.021	15
RTF2_HUMAN__MTBP_HUMAN__1210aa	1	1	71.2	0.289	10.6
RTF2_HUMAN__STAG2_HUMAN__1537aa	1	1	69.6	0.147	13.2
RTF2_HUMAN__ORC3_HUMAN__1017aa	1	1	67.8	0.067	12.7
RTF2_HUMAN__MCM10_HUMAN__1181aa	1	1	71.3	0.506	8.4
RTF2_HUMAN__STN1_HUMAN__674aa	1	1	71.7	0.034	15
RTF2_HUMAN__FANCG_HUMAN__928aa	1	1	68.9	0.044	11.8
RTF2_HUMAN__UBP1_HUMAN__1091aa	1	1	64.5	0.029	14.8
RTF2_HUMAN__SHLD2_HUMAN__1141aa	1	1	62.1	0.024	4.9
RTF2_HUMAN__TIM_HUMAN__1514aa	1	1	69	0.019	10
RTF2_HUMAN__RA51C_HUMAN__682aa	1	1	70.2	0.022	14.8

RTF2_HUMAN__MLH1_HUMAN__1062aa	1	1	77.4	0.394	13.9
RTF2_HUMAN__XPF_HUMAN__1222aa	1	1	67.4	0.038	10.5
RTF2_HUMAN__TEP1_HUMAN__2933aa	1	1	54	0.018	15
RTF2_HUMAN__DNA2_HUMAN__1366aa	1	1	70.7	0.045	9.7
RTF2_HUMAN__ERCC1_HUMAN__603aa	1	1	69.4	0.032	14
RTF2_HUMAN__REV1_HUMAN__1557aa	1	1	68.9	0.338	10.7
RTF2_HUMAN__UBXN7_HUMAN__795aa	1	2	82.8	0.461	11.1
RTF2_HUMAN__RAD9A_HUMAN__697aa	1	1	80.4	0.577	14.2
RTF2_HUMAN__CTF18_HUMAN__1281aa	1	1	82.8	0.632	3.2
RTF2_HUMAN__NSE1_HUMAN__572aa	1	1	78	0.041	14.8
RTF2_HUMAN__CA112_HUMAN__1159aa	1	1	73.2	0.322	8.6
RTF2_HUMAN__PDS5A_HUMAN__1643aa	1	1	73.8	0.234	8
RTF2_HUMAN__RAD52_HUMAN__724aa	1	1	70	0.29	11.5
RTF2_HUMAN__RFWD3_HUMAN__1080aa	1	1	72.3	0.159	14.4
RTF2_HUMAN__SMC3_HUMAN__1523aa	1	1	80.1	0.538	8.7
RTF2_HUMAN__CCNE1_HUMAN__716aa	1	1	68.5	0.031	12.1
RTF2_HUMAN__PSF1_HUMAN__502aa	1	1	68.9	0.254	12.5
RTF2_HUMAN__FIGL1_HUMAN__980aa	1	1	65.3	0.223	9.5
RTF2_HUMAN__ELOA1_HUMAN__1104aa	1	1	71.1	0.321	8.9
RTF2_HUMAN__SHLD1_HUMAN__511aa	1	1	72.8	0.057	14.7
RTF2_HUMAN__DPOLA_HUMAN__1768aa	1	1	75.3	0.225	7.9
RTF2_HUMAN__DTL_HUMAN__1036aa	1	1	77.3	0.063	14.7
RTF2_HUMAN__TERF1_HUMAN__745aa	1	1	77.2	0.117	14.8
RTF2_HUMAN__RNH2C_HUMAN__470aa	1	1	78.8	0.056	14.8
RTF2_HUMAN__TOPB1_HUMAN__1828aa	1	1	70.2	0.282	14.1

RTF2_HUMAN__ORC6_HUMAN__558aa	1	1	69	0.053	14.5
RTF2_HUMAN__HMCES_HUMAN__660aa	1	1	78.7	0.113	14.6
RTF2_HUMAN__CDC7_HUMAN__880aa	1	1	72.1	0.17	7.1
RTF2_HUMAN__GEMI_HUMAN__515aa	1	1	62.4	0.048	8
RTF2_HUMAN__PMS2_HUMAN__1168aa	1	1	68.8	0.08	6.9
RTF2_HUMAN__DPOD2_HUMAN__775aa	1	1	76.9	0.175	4.5
RTF2_HUMAN__DCR1A_HUMAN__1346aa	1	1	67.5	0.019	15
RTF2_HUMAN__ORC5_HUMAN__741aa	1	1	73.7	0.236	10.5
RTF2_HUMAN__EXO5_HUMAN__679aa	1	1	73.7	0.471	7.6
RTF2_HUMAN__DPOE4_HUMAN__423aa	1	1	72.2	0.098	14.2
RTF2_HUMAN__DPOA2_HUMAN__904aa	1	1	73.1	0.045	14.8
RTF2_HUMAN__CAF1A_HUMAN__1262aa	1	1	57	0.018	15
RTF2_HUMAN__DNLI1_HUMAN__1225aa	1	1	72.5	0.239	5.5
RTF2_HUMAN__RAD18_HUMAN__801aa	1	1	60.4	0.058	12.7
RTF2_HUMAN__FACD2_HUMAN__1757aa	1	1	70.1	0.024	15
RTF2_HUMAN__PRIPO_HUMAN__866aa	1	1	72.9	0.029	15
RTF2_HUMAN__POLH_HUMAN__1019aa	1	1	85.3	0.665	6.2
RTF2_HUMAN__DPOD1_HUMAN__1413aa	1	1	67.8	0.094	8.1
RTF2_HUMAN__SLX4_HUMAN__2140aa	1	1	70	0.234	9
RTF2_HUMAN__STAG1_HUMAN__1564aa	1	1	68.2	0.18	4.5
RTF2_HUMAN__RNH2B_HUMAN__618aa	1	1	79.8	0.475	5.5
RTF2_HUMAN__PCNA_HUMAN__567aa	1	1	60.8	0.022	10.9
RTF2_HUMAN__FANCI_HUMAN__1634aa	1	1	84	0.674	5.3
RTF2_HUMAN__POLI_HUMAN__1046aa	1	1	72.2	0.158	6
RTF2_HUMAN__MUS81_HUMAN__857aa	1	2	67.4	0.182	11.5

RTF2_HUMAN__MMS22_HUMAN__1549aa	0	0	0	0	0
RTF2_HUMAN__TOP3A_HUMAN__1307aa	0	0	0	0	0
RTF2_HUMAN__MSH3_HUMAN__1443aa	0	0	0	0	0
RTF2_HUMAN__MD2L2_HUMAN__517aa	0	0	0	0	0
RTF2_HUMAN__DBF4B_HUMAN__921aa	0	0	0	0	0
RTF2_HUMAN__MRE11_HUMAN__1014aa	0	0	0	0	0
RTF2_HUMAN__ESCO1_HUMAN__1146aa	0	0	0	0	0
RTF2_HUMAN__SPAT5_HUMAN__1199aa	0	0	0	0	0
RTF2_HUMAN__WDHD1_HUMAN__1435aa	0	0	0	0	0
RTF2_HUMAN__FAN1_HUMAN__1323aa	0	0	0	0	0
RTF2_HUMAN__TRAIP_HUMAN__775aa	0	0	0	0	0
RTF2_HUMAN__SLN11_HUMAN__1207aa	0	0	0	0	0
RTF2_HUMAN__SPA5L_HUMAN__1059aa	0	0	0	0	0
RTF2_HUMAN__NSE4A_HUMAN__691aa	0	0	0	0	0
RTF2_HUMAN__F111A_HUMAN__917aa	0	0	0	0	0
RTF2_HUMAN__CUL2_HUMAN__1051aa	0	0	0	0	0
RTF2_HUMAN__BLM_HUMAN__1723aa	0	0	0	0	0
RTF2_HUMAN__DMC1_HUMAN__646aa	0	0	0	0	0
RTF2_HUMAN__PDS5B_HUMAN__1753aa	0	0	0	0	0
RTF2_HUMAN__ESCO2_HUMAN__907aa	0	0	0	0	0
RTF2_HUMAN__ORC4_HUMAN__742aa	0	0	0	0	0
RTF2_HUMAN__ORC1_HUMAN__1167aa	0	0	0	0	0
RTF2_HUMAN__TCAB1_HUMAN__854aa	0	0	0	0	0
RTF2_HUMAN__MCMBP_HUMAN__948aa	0	0	0	0	0
RTF2_HUMAN__DPOD4_HUMAN__413aa	0	0	0	0	0

RTF2_HUMAN__DPOE1_HUMAN__2592aa	0	0	0	0	0
RTF2_HUMAN__TE2IP_HUMAN__705aa	0	0	0	0	0
RTF2_HUMAN__MCM7_HUMAN__1025aa	0	0	0	0	0
RTF2_HUMAN__TOP2A_HUMAN__1837aa	0	0	0	0	0
RTF2_HUMAN__FAP20_HUMAN__486aa	0	0	0	0	0
RTF2_HUMAN__NIPBL_HUMAN__3110aa	0	0	0	0	0
RTF2_HUMAN__CDC45_HUMAN__872aa	0	0	0	0	0
RTF2_HUMAN__SMC5_HUMAN__1407aa	0	0	0	0	0
RTF2_HUMAN__RFC2_HUMAN__660aa	0	0	0	0	0
RTF2_HUMAN__MCM4_HUMAN__1169aa	0	0	0	0	0
RTF2_HUMAN__FAF1_HUMAN__956aa	0	0	0	0	0
RTF2_HUMAN__RFC1_HUMAN__1454aa	0	0	0	0	0
RTF2_HUMAN__DCR1B_HUMAN__838aa	0	0	0	0	0
RTF2_HUMAN__FANCF_HUMAN__680aa	0	0	0	0	0
RTF2_HUMAN__RNH2A_HUMAN__605aa	0	0	0	0	0
RTF2_HUMAN__MCM6_HUMAN__1127aa	0	0	0	0	0
RTF2_HUMAN__MSH2_HUMAN__1240aa	0	0	0	0	0
RTF2_HUMAN__RA51D_HUMAN__634aa	0	0	0	0	0
RTF2_HUMAN__RFA3_HUMAN__427aa	0	0	0	0	0
RTF2_HUMAN__GEN_HUMAN__1214aa	0	0	0	0	0
RTF2_HUMAN__CDC6_HUMAN__866aa	0	0	0	0	0
RTF2_HUMAN__RAD50_HUMAN__1618aa	0	0	0	0	0
RTF2_HUMAN__DPOE3_HUMAN__453aa	0	0	0	0	0
RTF2_HUMAN__SHPRH_HUMAN__1989aa	0	0	0	0	0
RTF2_HUMAN__SP16H_HUMAN__1353aa	0	0	0	0	0

RTF2_HUMAN__RAD51_HUMAN__645aa	0	0	0	0	0
RTF2_HUMAN__MSH6_HUMAN__1666aa	0	0	0	0	0
RTF2_HUMAN__HUS1_HUMAN__586aa	0	0	0	0	0
RTF2_HUMAN__UBE2B_HUMAN__458aa	0	0	0	0	0
RTF2_HUMAN__FANCC_HUMAN__864aa	0	0	0	0	0
RTF2_HUMAN__PSF3_HUMAN__522aa	0	0	0	0	0
RTF2_HUMAN__FSCN1_HUMAN__799aa	0	0	0	0	0
RTF2_HUMAN__FANCM_HUMAN__2354aa	0	0	0	0	0
RTF2_HUMAN__SLF2_HUMAN__1479aa	0	0	0	0	0
RTF2_HUMAN__DPOD3_HUMAN__772aa	0	0	0	0	0
RTF2_HUMAN__FEN1_HUMAN__686aa	0	0	0	0	0
RTF2_HUMAN__PRI2_HUMAN__815aa	0	0	0	0	0
RTF2_HUMAN__MCM5_HUMAN__1040aa	0	0	0	0	0
RTF2_HUMAN__RAD21_HUMAN__937aa	0	0	0	0	0
RTF2_HUMAN__SMC6_HUMAN__1397aa	0	0	0	0	0
RTF2_HUMAN__SLX1_HUMAN__581aa	0	0	0	0	0
RTF2_HUMAN__MLH3_HUMAN__1759aa	0	0	0	0	0
RTF2_HUMAN__CDCA5_HUMAN__558aa	0	0	0	0	0
RTF2_HUMAN__RA54B_HUMAN__1216aa	0	0	0	0	0
RTF2_HUMAN__MSH4_HUMAN__1242aa	0	0	0	0	0
RTF2_HUMAN__NSE2_HUMAN__553aa	0	0	0	0	0
RTF2_HUMAN__NEIL3_HUMAN__911aa	0	0	0	0	0
RTF2_HUMAN__DBF4A_HUMAN__980aa	0	0	0	0	0
RTF2_HUMAN__PSF2_HUMAN__491aa	0	0	0	0	0
RTF2_HUMAN__ACD_HUMAN__764aa	0	0	0	0	0

RTF2_HUMAN__FANCJ_HUMAN__1555aa	0	0	0	0	0
RTF2_HUMAN__LLR1_HUMAN__720aa	0	0	0	0	0
RTF2_HUMAN__FAP24_HUMAN__521aa	0	0	0	0	0
RTF2_HUMAN__RFA2_HUMAN__576aa	0	0	0	0	0
RTF2_HUMAN__ZRAB3_HUMAN__1385aa	0	0	0	0	0
RTF2_HUMAN__CTIP_HUMAN__1203aa	0	0	0	0	0
RTF2_HUMAN__RECQ1_HUMAN__955aa	0	0	0	0	0
RTF2_HUMAN__PALB2_HUMAN__1492aa	0	0	0	0	0
RTF2_HUMAN__TTF2_HUMAN__1468aa	0	0	0	0	0
RTF2_HUMAN__FANCA_HUMAN__1761aa	0	0	0	0	0
RTF2_HUMAN__RFC4_HUMAN__669aa	0	0	0	0	0
RTF2_HUMAN__NEIL1_HUMAN__696aa	0	0	0	0	0
RTF2_HUMAN__MCM3_HUMAN__1114aa	0	0	0	0	0
RTF2_HUMAN__DCC1_HUMAN__699aa	0	0	0	0	0
RTF2_HUMAN__UBP37_HUMAN__1285aa	0	0	0	0	0
RTF2_HUMAN__FP100_HUMAN__1187aa	0	0	0	0	0
RTF2_HUMAN__TOP2B_HUMAN__1932aa	0	0	0	0	0
RTF2_HUMAN__CTC1_HUMAN__1523aa	0	0	0	0	0
RTF2_HUMAN__RAD1_HUMAN__588aa	0	0	0	0	0
RTF2_HUMAN__RAD17_HUMAN__987aa	0	0	0	0	0
RTF2_HUMAN__POTE1_HUMAN__940aa	0	0	0	0	0
RTF2_HUMAN__SLD5_HUMAN__529aa	0	0	0	0	0
RTF2_HUMAN__MCM9_HUMAN__1449aa	0	0	0	0	0
RTF2_HUMAN__CLSPN_HUMAN__1645aa	0	0	0	0	0
RTF2_HUMAN__NBN_HUMAN__1060aa	0	0	0	0	0

RTF2_HUMAN__BARD1_HUMAN__1083aa	0	0	0	0	0
RTF2_HUMAN__SMC1A_HUMAN__1539aa	0	0	0	0	0
RTF2_HUMAN__DYL1_HUMAN__395aa	0	0	0	0	0
RTF2_HUMAN__CTF8_HUMAN__427aa	0	0	0	0	0
RTF2_HUMAN__RFC5_HUMAN__646aa	0	0	0	0	0
RTF2_HUMAN__CAF1B_HUMAN__865aa	0	0	0	0	0
RTF2_HUMAN__REV3L_HUMAN__3436aa	0	0	0	0	0
RTF2_HUMAN__RAD54_HUMAN__1053aa	0	0	0	0	0
RTF2_HUMAN__FANCE_HUMAN__842aa	0	0	0	0	0
RTF2_HUMAN__DPOLQ_HUMAN__2896aa	0	0	0	0	0
RTF2_HUMAN__CDT1_HUMAN__852aa	0	0	0	0	0
RTF2_HUMAN__XRCC2_HUMAN__586aa	0	0	0	0	0
RTF2_HUMAN__ATRIP_HUMAN__1097aa	0	0	0	0	0
RTF2_HUMAN__HLTF_HUMAN__1315aa	0	0	0	0	0
RTF2_HUMAN__MDC1_HUMAN__2395aa	0	0	0	0	0
RTF2_HUMAN__RTEL1_HUMAN__1525aa	0	0	0	0	0
RTF2_HUMAN__CIP2A_HUMAN__1211aa	0	0	0	0	0
RTF2_HUMAN__TICRR_HUMAN__2216aa	0	0	0	0	0
RTF2_HUMAN__RFA1_HUMAN__922aa	0	0	0	0	0
RTF2_HUMAN__XRCC3_HUMAN__652aa	0	0	0	0	0
RTF2_HUMAN__PINX1_HUMAN__634aa	0	0	0	0	0
RTF2_HUMAN__DPOE2_HUMAN__833aa	0	0	0	0	0
RTF2_HUMAN__TEN1L_HUMAN__429aa	0	0	0	0	0
RTF2_HUMAN__CDK2_HUMAN__604aa	0	0	0	0	0
RTF2_HUMAN__SLF1_HUMAN__1364aa	0	0	0	0	0

RTF2_HUMAN__ATR_HUMAN__2950aa	0	0	0	0	0
RTF2_HUMAN__RECQ4_HUMAN__1514aa	0	0	0	0	0
RTF2_HUMAN__WRN_HUMAN__1738aa	0	0	0	0	0
RTF2_HUMAN__MSH5_HUMAN__1140aa	0	0	0	0	0
RTF2_HUMAN__SPRTN_HUMAN__795aa	0	0	0	0	0
RTF2_HUMAN__MCM2_HUMAN__1210aa	0	0	0	0	0
RTF2_HUMAN__RA51B_HUMAN__690aa	0	0	0	0	0
RTF2_HUMAN__HELQ_HUMAN__1407aa	0	0	0	0	0
RTF2_HUMAN__TERT_HUMAN__1438aa	0	0	0	0	0
RTF2_HUMAN__MCM8_HUMAN__1146aa	0	0	0	0	0
RTF2_HUMAN__PMS1_HUMAN__1238aa	0	0	0	0	0
RTF2_HUMAN__DONS_HUMAN__872aa	0	0	0	0	0
RTF2_HUMAN__FANCB_HUMAN__1165aa	0	0	0	0	0
RTF2_HUMAN__EXO1_HUMAN__1152aa	0	0	0	0	0
RTF2_HUMAN__POLK_HUMAN__1176aa	0	0	0	0	0
RTF2_HUMAN__EME2_HUMAN__685aa	0	0	0	0	0
RTF2_HUMAN__TIPIN_HUMAN__607aa	0	0	0	0	0

Table S2. Key resource table

REAGENT OR RESOURCE	SOURCE	IDENTIFIER
Antibodies		
Rabbit monoclonal anti-phospho-FOXM1 (Thr600), WB/IF: 1:1000	Cell Signaling Technology, Cat#14655	RRID:AB_2798557
Mouse monoclonal anti-vinculin, Unconjugated, Clone hVIN-1, WB: 1:5000	MilliporeSigma, Cat#V9131	RRID:AB_477629
Mouse monoclonal anti-cyclin B1 (D-11), IF: 1:1000	Santa Cruz Biotechnology, Cat#sc-7393	RRID:AB_627336
Mouse monoclonal anti-CDC45 (G-12), IF: 1:1000	Santa Cruz Biotechnology, Cat#sc-55569	RRID:AB_831146
Rabbit monoclonal anti-ETAA1 (E8u8B), IF: 1:1000	Cell Signaling Technology, Cat#77021	N/A
Rabbit polyclonal anti-TRESLIN, IF: 1:1000	Bethyl Cat#A303-472A	RRID:AB_10953949
Rabbit monoclonal anti-HA tag, WB/IF: 1:1000	Cell Signaling Technology, Cat#C29F4	RRID:AB_1549585
Mouse monoclonal anti-CHK1 (2G1D5), WB/IF/PLA: 1:1000	Cell Signaling Technology Cat#2360	RRID:AB_2080320
Rabbit polyclonal anti-RTF2, WB/IF/PLA: 1:1000	ProteinTech Cat#16633-1-AP	RRID:AB_2256547
Rabbit monoclonal anti-MCM7 (D10A11) XP, WB: 1:1000	Cell Signaling, Cat#3735 S	RRID:AB_2142705
Mouse monoclonal anti-PCNA (PC10), WB: 1:1000	Santa Cruz, Cat#sc-56	RRID:AB_628110
Goat polyclonal anti-MCM6 (C-20), WB: 1:1000	Santa Cruz Biotechnology Cat#sc-9843	RRID:AB_2142543
Mouse monoclonal anti-PCNA (PC10), WB: 1:1000	Santa Cruz, Cat#sc-56	RRID:AB_628110
Mouse monoclonal anti-CLASPIN (B-6), WB/IF: 1:500, PLA: 1:1000	Santa Cruz, Cat#sc-376773	RRID:AB_3697838
Mouse monoclonal anti-FLAG (M2), IF: 1:1000	Sigma-Aldrich Cat#F1804	RRID:AB_262044
Rabbit polyclonal anti-c20orf43 (RTF2), IF: 1:1000	Novus, Cat#NBP2-30645	N/A

Mouse monoclonal anti-biotin, PLA: 1:1000	Jackson ImmunoResearch, Cat#200-002-211	RRID:AB_2339006
Mouse monoclonal anti-BrdU (B44), Combing: 1:10	BD Biosciences, Cat#347580	RRID:AB_400326
Rat monoclonal anti-BrdU [BU1/75 (ICR1)], Combing: 1:20	Abcam, Cat#ab6326	RRID:AB_305426
Bacterial strains		
DH5-α *E. coli*	New England Biolabs	Cat#C2987
NEB Stable Competent *E. coli*	New England Biolabs	Cat#C3040
Chemicals, peptides, and recombinant proteins		
TransIT-293	MirusBio LLC	Cat# MIR2700
Lipofectamine RNAiMAX Transfection Reagent	ThermoFisher	Cat# 13778-150
CDK1/2i (Cdk1/2 Inhibitor III)	Millipore Sigma	Cat# 217714
CDC7i (Simurosertib/TAK-931)	MedChemExpress	Cat#HY-100888
ATRi (AZ20)	Selleck Chemicals	Cat#S2683
CHK1i (CHIR-124)	Abcam	Cat#ab273615
ATMi (KU-55933)	Selleck Chemicals	Cat#S1092
DNA-PKcsi (AZD7648)	Selleck Chemicals	Cat#S8843
V-1 (dTAG^{V}-1)	bio-techne TOCRIS	Cat#6914
5'-cholor-2'-deoxyuridine (CldU)	MilliporeSigma	Cat# C6891
5-ethynyl-2'-deoxyuridine (EdU)	ThermoFisher	Cat# A10044
AZDye 488 Picolyl Azide	Vector Laboratories	Cat#CCT-1276
5'-iodo-2'-deoxyuridine (IdU)	MilliporeSigma	Cat# I7125
cOmplete™, EDTA-free Protease Inhibitor Cocktail	MilliporeSigma	Cat# 11873580001
Phosphatase Inhibitor Cocktail I	MilliporeSigma	Cat# 524624
Phosphatase Inhibitor Cocktail II	MilliporeSigma	Cat# 524625
Benzonase® Nuclease	Sigma Aldrich	E1014
Critical commercial assays		
DC™ Protein Assay	Bio-Rad	Cat# 5000111

DuoLink® In Situ PLA Anti-mouse MINUS probe	MilliporeSigma	Cat# DUO92004
DuoLink ®In Situ PLA Anti-rabbit PLUS probe	MilliporeSigma	Cat# DUO92002
DuoLink® In Situ Detection Reagents Orange	MilliporeSigma	Cat# DUO92007
DuoLink® In Situ Wash Buffers	MilliporeSigma	Cat# DUO82049
Experimental models: Cell lines		
Human: HEK293T	ATCC, Cat# CRL-3216	RRID:CVCL_0063
HeLa (Parental, RNase H2A knockout (KO))	Durocher and Jackson Labs	(*43*)
Mouse: RTF2 MEFs (*Rtf2*$^{-/lox}$, SV40-immortalized *Rtf2*$^{-/-}$ clone, *Rtf2*$^{-/lox}$*;Trp53*$^{-/-}$)	Smogorzewska Lab	(*20*)
Oligonucleotides		
RTF2^{4N} geneblock	See Methods for sequence	This paper
Recombinant DNA		
pDONR223; spectinomycin resistant; Gateway	Invitrogen	Vidal Lab
pENTR-EV; spectinomycin resistant	Made by Jianping Jin	Elledge Lab
pENTR-hRTF2; spectinomycin resistant	This paper	This paper
pENTR-hRTF2^{4N} (S275N; A277N; Y278N; S280N)	This paper	This paper
pMMP Hit & Run Cre; retroviral; self-excising	Livingston Lab	Livingston Lab (*44*)
pDEST-MSCV_IP-N-HA/Flag; Gateway destination vector	Made by Jianping Jin	Elledge Lab
MSCV_IP-N-HA/FLAG-EV	Made by Jianping Jin	Elledge Lab
MSCV_IP-N-HA/FLAG-hRTF2	This paper	This paper
MSCV_IP-N-HA/FLAG-hRTF2^{4N} (S275N; A277N; Y278N; S280N)	This paper	This paper
Claspin Mission shRNA CLASPIN #1	Sigma-Aldrich	TRCN0000175992
Claspin Mission shRNA CLASPIN #2	Sigma-Aldrich	TRCN0000193398
pLKO.1 shRNA Control	MilliporeSigma	SHC002
pLEX_305-N-dTAG	Addgene	#91797
pLEX_305-N-HA-dTAG-RTF2	This paper	This paper
pLEX_305-N-HA-dTAG-RTF2^{PAM} (c.346C>A; c.348G>A)	This paper	This paper

pLEX_305-N-HA-dTAG-RH2A	This paper	This paper
CHK1-IRES-GFP	Precision Lenti ORFeome	
VSV-G; retroviral packaging		
Gagpol; retroviral packaging		
pMD2.G (VSV-G envelope expressing plasmid); lentiviral packaging	Addgene	#12259
psPAX2; lentiviral packaging	Addgene	#12260
Software and algorithms		
Prism	GraphPad	Version 10.4.2
CellProfiler	Broad Institute	Version 4.2.6(*45*)
FIJI	Cardona Lab	Version 2.14.0(*46*)
Other		
Silencer Select RTFDC1	siRNA ID: s226737	Cat#4392420
ON-TARGETplus Human CHEK1 siRNA SMARTpool	Horizon Discovery	Cat# L-003255-00-0005
Silencer Select CLSPN	siRNA IDs s34330, s34331, s34332	Cat# 4392420
ON-TARGETplus Human ETAA1 siRNA SMARTpool	Horizon Discovery	Cat# L-021193-01-0005
ON-TARGETplus Human TICRR (TRESLIN) siRNA SMARTpool	Horizon Discovery	L-018755-02-0005
